# Dwarna: a blockchain solution for dynamic consent in biobanking

**DOI:** 10.1038/s41431-019-0560-9

**Published:** 2019-12-16

**Authors:** Nicholas Mamo, Gillian M. Martin, Maria Desira, Bridget Ellul, Jean-Paul Ebejer

**Affiliations:** 1 Centre for Molecular Medicine and Biobanking, Biomedical Sciences Building, University of Malta, Msida, MSD 2080 Malta; 2 Department of Sociology, Faculty of Arts, University of Malta, Msida, MSD 2080 Malta; 3 BBMRI-ERIC, Neue Stiftingtalstraße 2/B/6, 8010 Graz, Austria; 4 Department of Pathology, Faculty of Medicine and Surgery, University of Malta, Msida, MSD 2080 Malta

**Keywords:** Medical research, Social sciences

## Abstract

Dynamic consent aims to empower research partners and facilitate active participation in the research process. Used within the context of biobanking, it gives individuals access to information and control to determine how and where their biospecimens and data should be used. We present Dwarna—a web portal for ‘dynamic consent’ that acts as a hub connecting the different stakeholders of the Malta Biobank: biobank managers, researchers, research partners, and the general public. The portal stores research partners’ consent in a blockchain to create an immutable audit trail of research partners’ consent changes. Dwarna’s structure also presents a solution to the European Union’s General Data Protection Regulation’s right to erasure—a right that is seemingly incompatible with the blockchain model. Dwarna’s transparent structure increases trustworthiness in the biobanking process by giving research partners more control over which research studies they participate in, by facilitating the withdrawal of consent and by making it possible to request that the biospecimen and associated data are destroyed.

## Introduction

Trust is a major pillar in the relational dynamics within the process of genomic research. This field requires robust and transparent consenting procedures to be ethical, and only through consistently being ethical can trust be achieved. In fact, Lipworth, Forsyth, and Kerridge describe winning trust as a gradual process—*“an emergent property of good social relationships that are built over time”* [1].

Biobanking sees biobank managers collect or curate biospecimens, such as DNA, from the general public or from individuals affected by a specific disease. These biospecimens are stored for long-term use in the biobank, alongside links to personal health and lifestyle data to be used by researchers in scientific studies [1].

For this reason, biobanking for genomic research has complex ethical, legal, and social implications [2]. Informed consent is the backbone underlying participation in research, ensuring the practice of ethical principles in line with the Helsinki Declaration [3]. Informed consent ‘protects the individual’s freedom of choice and respects the individual’s autonomy’ [4], thus safeguarding the fundamental rights of human dignity and integrity [5, 6]. The process of recruitment in biobanking, and engagement in research participation can be improved if consent is not a one-off event but is sustained throughout the research, a process called dynamic consent [7].

Dynamic consent is one protocol of consent that is ethical and complies with the law and regulations [2, 8]. It aims to give individuals the opportunity to be better informed about their consent choices and the ongoing research process in general, and gives them control over how their biospecimens and data are used. This process can be facilitated if dynamic consent is available on a digital platform, which can also be used to register interest in participating in new research projects. In this way, dynamic consent sees individuals not simply as research participants, but as research partners who make their own decisions on how to participate.

At the Malta Biobank, we have now chosen to consistently apply the term ‘research partner’ for individuals providing a biospecimen for current or future research. Although this term is not yet in uniform use, we strongly believe that dynamic consent is a tool that permits *“understanding and supporting biomedical research as a partnership between participants and researchers”* [2] and places research participants *“at the centre of decision making as equal partners in the research process”* [9]. Thus, genuine participation in participant-centered research initiatives will hopefully result in research participants being *“not just subjects for research and interventions, but instead partners”* [10].

In this paper, we present Dwarna, a web portal for dynamic consent that harnesses the blockchain model. The term Dwarna—‘about us’ in Maltese—represents the project’s objective of helping genomic research to gather data about humans, with the ultimate aim of improving healthcare for the wider community.

The project is hosted at the Centre for Molecular Medicine and Biobanking at the University of Malta, which itself hosts the Malta Biobank [11]—the Maltese national node of BBMRI-ERIC [12]. The Dwarna portal will act as a hub connecting the biobank managers, researchers, research partners, and the general public. Dwarna has a data controller who ensures that the portal operates in compliance with the General Data Protection Regulation (GDPR) so as to safeguard research partners. The data controller of Dwarna is the same individual as the data controller of the Malta Biobank, who is also the biobank manager.

In Dwarna, we use the blockchain for its immutable nature, storing in it the research partners’ consent changes more transparently. However, the blockchain is perceived as being in conflict with the European Union’s recently introduced GDPR, which encourages data minimization and gives citizens the right to erasure (also known as the right to be forgotten) [13]. The right to erasure gives individuals the right to request that the data that an institution stores about them are destroyed. Thus, we present a solution that overcomes these challenges to comply with the regulation and ensure the rights of individuals are securely protected.

In this paper, we first explore existing systems for obtaining consent in the field of genomic research and the blockchain. Subsequently, we present Dwarna’s architecture. Finally, we discuss how Dwarna contributes a more trustworthy solution to biobanking.

## Related work

This section provides an insight into how dynamic consent can potentially make biobanking, and subsequently genomic research, more efficient and transparent. This section also explores the blockchain and existing applications in healthcare and genomic research.

### Biobanking and informed consent

From minimizing financial burdens [14] to gathering comprehensive medical histories [15], data sharing in healthcare presents a multitude of benefits. When framed within the context of rare diseases, it is clear that linkages between datasets across research centers are key to progress in research [16]. This said, sharing sensitive data is also a process that incurs risks, not least the risk of losing control over this data. Today, control is not only desirable [17], but demanded by the research partners themselves [8, 18, 19] and emphasized in the European Union’s GDPR, where recital 7 states that *“natural persons should have control of their own personal data”* [20].

In the field of medical research, biobanking relies on individuals sharing their biospecimens and data with biobanks. Biobank managers collect biological samples, such as blood, tissues, cells, and DNA, and associated health and lifestyle data from research partners, store them and make them available to researchers for use in scientific research projects [21]. In the past, there have been two key approaches for the consenting procedure for biobank-based research.

The most common approach is broad consent whereby research partners give consent for the use of their biospecimens and data, sometimes with few specific restrictions [22], or none at all [23]. This enables their reuse for future, as yet unspecified, research projects without the need of being re-contacted or consulted. Whereas broad consent does facilitate the optimal use of the biospecimens and associated data, it also comes at the expense of participant engagement and control, which may adversely affect willingness to take part in research. The alternative is a one-time informed consent for a specific research project, which significantly limits the possibility of using the biospecimen and its data to their maximum potential in other research studies [23].

While offering the best potential for reuse of research partners’ biospecimens and data, broad consent is also associated with ethical lacunae. It restricts autonomy in decision making as to whether the aims and risks of a new research project are acceptable, though specific limitations can be inbuilt to limit this, particularly by ensuring *“oversight and approval of future research activities”* and *“an ongoing process of providing information”* [22]. Research has found that *“the more unclear the purpose of the sample and data usage is, the less there is a trust in the ‘appropriateness’ of research”* [24]. The key issue is the ambiguity of ‘informed consent’. Informed consent expects researchers to provide research partners with details of how their sample shall be used, yet the future of biotechnology is unanticipatable and thus the potential future uses of biospecimens or data are unknown [21]. More recently, a new alternative, delivered through digital technology, has emerged—dynamic consent [2].

Dynamic consent does not automatically exclude broad consent. Instead, it is a new and enhanced option of informed consent that gives research partners greater control over the extent of their participation, providing the possibility of re-considering consent to participate at different stages of a research project, allowing any arising ethical issues to be addressed. This is particularly likely in the ever-developing field of genomic research. Due to potential future developments in genomic research, the exclusive use of one-time consent is questionable if ethical standards are to be preserved. Since biobanks store biospecimens for decades, one-time consent cannot be regarded as informed consent, as new, unforeseen, biotechnologies are bound to be invented. Thus, what is required is an ongoing process of consent which reflects these developments [2].

Kaye et al. [2] further claim that dynamic consent enhances respect for research partners by *“giving individuals as much choice and control in what is done with their personal information and material as is reasonably achievable.”* Simultaneously, the practice of pseudonymization and data encryption safeguards confidentiality.

Dynamic consent is normally delivered via an IT interface that serves as a communication system between stakeholders; namely the biobank managers and researchers at one end, and the research partners at the other end. In this way, existing dynamic consent implementations, such as those by the NHS [25] or RUDY [8, 26] in the UK, and the Cooperative Health Research in South Tyrol (CHRIS) [27, 28] allow research partners to become active members in the research process [2].

With dynamic consent, research partners may give consent to participate in ongoing projects and may also withdraw consent at any point. Thus, research partners enjoy greater control over their biospecimens and personal data whilst remaining in an ongoing relationship with biobanks [2]. Although research partners reserve the right to withdraw consent or ask for their biospecimen and data to be destroyed, this only applies to future uses, and does not affect already-processed data. Research partners who withdraw consent after their data have been processed shall not have their biospecimen reused and any link between the data and the biospecimen is destroyed. Nevertheless, already-processed data can still be used as its removal is usually not possible and would hinder research [29].

By giving research partners more control, dynamic consent also involves individuals in the decision-making process of genomic research. Research conducted by Robinson et al. [30] revealed that the vast majority of individuals participating in genomic research prefer to be involved in the decision-making process of data sharing among researchers. In turn, such a dynamic consent system keeps them better informed and provides transparency.

Most participants in genomic research are likely to accept data sharing if they are well-informed [31, 32]. Data sharing is at the core of best practice in genomic research as it allows for the maximum exploitation of the available data. Reluctance to share data often implies that the ultimate motive is personal accomplishment and not the common good [1]. Thus, ethically-sound genomic research calls for data sharing and reuse in different scientific research [33].

Dynamic consent is also ideal for improving trustworthiness [2]. Potentiating trustworthiness of biobanking activity in the eyes of the lay public and patient communities has been clearly identified in literature as being the key to ethical sustainability [34–38]. The key to building this trust is developing a system of governance based on accountability, transparency, and user control, which accommodates and protects the needs and rights of all stakeholders: research partners, researchers, and political or private sponsors [34, 35]. Such a goal can only be achieved by the use of transparent governance procedures [33].

Transparency protects the functioning of genomic research. Research shows that when research partners are informed and are offered a choice about what happens with their data, they are more likely to trust researchers, and consequently more likely to give consent for the use of their genetic data, as they perceive researchers as accountable and reliable [30].

Although transparency is dependent on the biobank’s functioning, laws and regulations like the GDPR heap responsibility on entities within the European Union to protect research partners’ personal data. Nevertheless, the technological solutions that host dynamic consent evoke concerns of privacy [39, 40] and security [39]. In healthcare, the blockchain model is emerging as one solution to these issues by contributing transparency and accountability [41].

### Blockchain

The blockchain model owes its origin to a 2008 white paper by Satoshi Nakamoto, who portrayed the blockchain as a network with a financial application—the Bitcoin cryptocurrency. Fundamentally, the blockchain is a distributed, immutable ledger—blocks of transactions chained together and shared by all nodes in the blockchain network [42].

In Nakamoto’s original proposal, nodes, or peers, traded Bitcoin among each other using public-key cryptography. To construct transactions, peers have a pair of public and private keys; the former is known by all nodes in the blockchain, whereas the latter is kept secret.

Peers use the private key to digitally sign their transactions. This digital signature can be decrypted using the paired public key, which yields the transaction contents. However, if someone maliciously tampers with the transaction, the transaction would not match with the decrypted digital signature. Thus, digital signatures make blockchain transactions tamper-proof.

The blockchain broadcasts all transactions to some nodes known as miners. Miners validate transactions, group them into a block and find a solution to a mathematical challenge to commit the block to the blockchain in return for a reward.

**Fig. 1:**
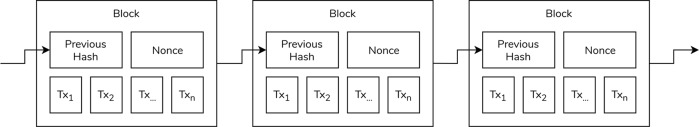
The typical blockchain structure. Figure adapted from Nakamoto [42].

The challenge incorporates this block of transactions and information from the previous block, as shown in Fig. 1. The reference to the previous block is its hash—a static-sized representation of the block’s contents that effectively cannot be reversed. The solution to the challenge is a number, known as a nonce. This nonce acts as proof-of-work and is meant to be easily-verifiable.

The blockchain uses the proof-of-work consensus mechanism to secure the chain’s integrity; maliciously tampering with one intermediate block changes its hash, which acts as the link with the next block. Therefore, the change invalidates the proof-of-work of all ensuing blocks, which have to be recalculated. Since only the longest blockchain is ever accepted as valid, Nakamoto shows how reworking the proof-of-work of these blocks transforms malicious intent into a chase to catch up with the other, honest miners.

**Fig. 2:**
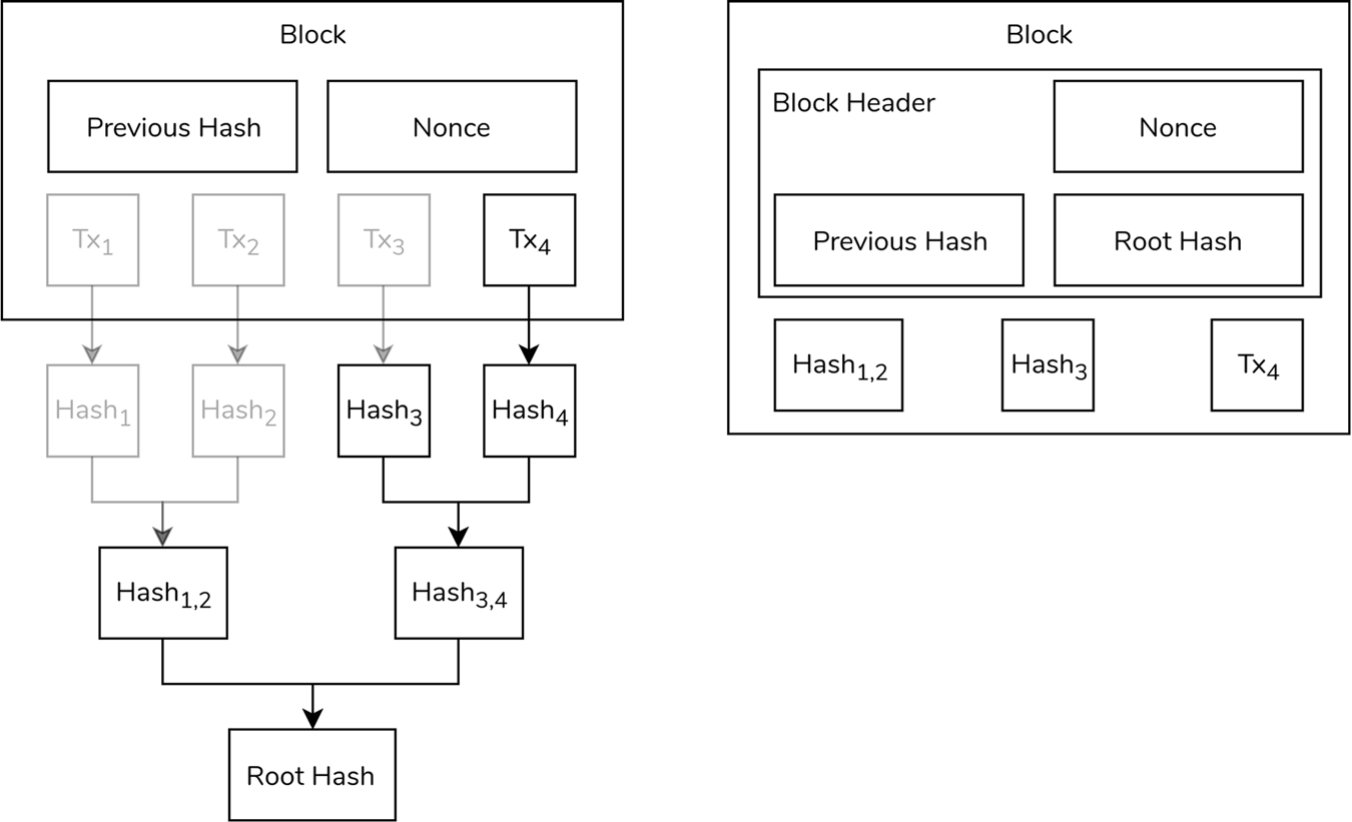
The block structure. **a** The calculation of the Merkle Root with pruned elements made transparent and **b** the block header after removing unnecessary data. Figure adapted from Nakamoto [42].

As the blockchain extends, old transactions and their Bitcoin are more likely to have been spent, rendering them useless. Therefore, old transactions need not be retained. Nakamoto’s solution is to replace the blocks with simpler block headers that replace transactions with the Merkle Root. The Merkle Root is constructed from the Merkle Tree—a binary tree with transactions as leaves. To get to the Merkle Root, the transactions are hashed. Subsequently, climbing up the tree, pairs of hashes are hashed again until only one hash remains—the Merkle Root, as shown in Fig. 2a).

Furthermore, not all hashes need to be retained to recalculate the Merkle Root. In the example of Fig. 2b), Transaction_4_, Hash_3_, and Hash_1,2_ are enough to calculate the Merkle Root and thus verify a block’s integrity. The Merkle Root is stored in the block’s header, alongside the necessary transactions and hashes, cutting down on storage space. More importantly, even with this limited data, the block’s hash is not broken.

Although the proof-of-work helps secure the blockchain’s integrity, it forces miners to compete to add blocks to the blockchain. The redundant efforts of miners results in extra energy overhead. Thus, other consensus mechanisms have been proposed. For example, in the proof-of-stake, the miner who adds a block to the blockchain is chosen based on how much cryptocurrency they can prove to hold [43]. These consensus mechanisms make it difficult to retrospectively alter the blockchain, practically making it immutable, and thus an alluring tool for various sectors, including healthcare.

Whereas Nakamoto’s Bitcoin’s blockchain has a narrow financial focus, the wide array of the blockchain’s applications has necessitated the creation of other digital ledgers. One such popular blockchain is Ethereum [34].

Although the principles of the blockchain remain, Ethereum’s scope is wider with its smart contracts, or self-executing code that can represent arbitrary applications. In this way, smart contracts bring applications from different industries into one blockchain. As a result, instead of having one cryptocurrency for each application, all of these smart contracts trade with the same cryptocurrency—ether.

Although a public Ethereum blockchain exists, it is also open-source, and can be downloaded and hosted privately. With the blockchain being used in more sensitive environments, including healthcare, as discussed later, restricting participant access to view or modify the ledger is imperative.

For this reason, other blockchain frameworks offer the possibility of hosting the blockchain privately, opening it up only to a select group of nodes. Hyperledger Composer [35], itself based on the Hyperledger Fabric blockchain framework [36], is one such solution.

## Hyperledger Composer

Hyperledger Fabric is an open-source framework for private, or permissioned, blockchains developed initially by the Linux Foundation, and later supported by companies such as IBM. As a blockchain framework, Hyperledger Fabric is immutable, with a two-piece ledger made up of a transaction log, akin to the more traditional ledger, and a current world state. Having an always-updated world state makes querying much more efficient in Hyperledger Fabric than in other blockchains.

Moreover, unlike Ethereum, Hyperledger Fabric has no cryptocurrency associated with it. In blockchains like Ethereum, cryptocurrency serves as an incentive for miners. Without cryptocurrency, the intensive proof-of-work scheme is unnecessary because there are no rewards to be won by miners. Thus, Hyperledger Fabric affords a more efficient scheme to add blocks to the ledger, as described next.

When network participants make a transaction, the involved peers endorse it to create a proposal. Other peers in the network, called ‘endorsing peers’ and defined by the blockchain’s endorsement policy, validate the transaction. They also execute the transaction against the ledger to get the updated state, although the ledger itself is not updated at this point. The endorsing peers return responses with the values of the executed transaction.

If the responses differ, potentially indicating nondeterminism, the transaction is rejected. If all returned responses are the same, these responses are sent to an ordering service, which orders responses chronologically into blocks of transactions. These blocks are broadcast to all peers in the blockchain, which validate the transactions and update their own ledger.

Like Ethereum, Hyperledger Fabric adopts the concept of smart contracts, or chaincode. Hyperledger Composer facilitates developing the chaincode through the business network definition. This definition includes assets, or items that are traded in the blockchain network; participants that own or modify these assets; and the transactions that effect these changes and form blocks in the blockchain.

To facilitate development, Hyperledger Composer can serve REST APIs to manage the network model. REST APIs can either be created in single or multiple user mode. In the former case, all transactions are signed by one user, whereas in the latter case the network participants sign their own transactions.

In both cases, participants have their own identity that is used to sign transactions, which is packaged in a business network card. In multiple user mode, these cards are used by participants to make their own REST API requests, rather than have the administrator make all requests on their behalf, thereby retaining accountability. The initial endpoints allow management of assets and participants. However, Hyperledger Composer provides a query language to extend the REST API with custom requests.

To identify network participants, Hyperledger Composer uses an authentication middleware—Passport.js [44]. Participants must first authenticate themselves with Passport.js using a service, such as Google. When they do so, Hyperledger Composer gives the participants an access token.

At this point, Hyperledger Composer also makes a local wallet available to store business network cards. Hyperledger Composer creates business network cards when it issues identities for participants in the blockchain. Initially, these cards lack credentials, but when imported into a wallet and “pinged”, the network adds credentials to the cards, allowing further reuse. Thus, whenever a network participant makes a transaction in the blockchain using the REST API, they sign the transaction themselves.

Whereas Hyperledger Fabric uses channels to restrict data sharing in the blockchain, Hyperledger Composer has its own access control language. This language defines who has access to view or modify assets in the blockchain network. The access control languages also propagate to queries.

### Blockchain in healthcare

A short three years after Nakamoto’s white paper, Estonia had already partnered with the private sector to start storing medical records in the blockchain [45]. Since then, more use cases of the blockchain in healthcare have emerged in literature, although few implementations are available. In most cases, giving patients control over their data remains a priority to instill trust. A literature selection of existing and proposed systems is provided in Table 1.

**Table 1:** Existing or proposed applications in healthcare and medical research that use the blockchain.

Reference	Description	Consent	Data deletion	Accessibility	Primary data storage	Technology	Stage	Availability
Dwarna (described in this article)	Dwarna is a web portal for ‘dynamic consent’ that acts as a hub connecting the different stakeholders of the Malta Biobank—biobank managers, researchers, research participants, and the general public. The portal stores research participants’ consent in a blockchain to create an immutable audit trail of research participants’ consent. Dwarna’s structure also presents a solution to the European Union’s General Data Protection Regulation (GDPR)’s right to be forgotten—a right that is seemingly incompatible with the blockchain model.	Dynamic	Yes	Private	Mixed	Hyperledger Composer	Prototype	GitHub
Al Omar et al. [47]	MediBchain is a protocol wherein the blockchain stores encrypted healthcare data. Users register with the system, authenticate themselves and send encrypted healthcare data to the blockchain. Blockchain transactions return an identifier or reference.	Not applicable	No		Blockchain		Framework/Protocol	
Chen et al. [54]	A proposed system that focuses on the secure storage of medical records, which are stored off-chain. The blockchain indexes these records. Hashing is used to ensure data integrity.	Dynamic	Yes		Off-chain		Proposal/Design	
Choudhury et al. [29]	A proposed system of dynamic consent to be compliant with the IRB regulations on data collection, with a focus on human research issues, including those encountered by biobanks. The proposed system would be hosted on a Hyperledger Fabric blockchain.	Dynamic	Yes	Private	Off-chain	Hyperledger Fabric	Proposal/Design	
Cyran [58]	An Ethereum-based solution that stores references to healthcare data distributed among many nodes off-chain. Patients own data, and they can share it with designated users and revoke that permission later. The goal is to be able to deploy this system to a hospital to enable healthcare data sharing between patients and healthcare professionals.	Dynamic	Yes	Consortium	Off-chain	Ethereum		
Dey et al. [65]	A system wherein a sensor is attached to a patient's bed and communicates with the IoT platform. This platform uses a REST API to manage the healthcare data that are collected by the sensors. All data are stored on the blockchain.	Not specified	No		Blockchain			
Dubovitskaya et al. [56]	A prototype for sharing EHR aimed at sharing patient information among hospitals and aggregating data among researchers on a Hyperledger Fabric blockchain.	Grant only	Yes	Private	Off-chain	Hyperledger Fabric	Prototype	
Ekblaw et al. [46]	Medrec permits patients to share healthcare data with clinicians and revoke permission later. The Ethereum blockchain stores these permissions. The data are stored off-chain. Miners—clinicians—are rewarded by aggregate data. The implementation is available on GitHub.	Dynamic	Yes		Off-chain	Ethereum	Implementation	GitHub
Faber et al. [59]	Blockchain-based Personal Data and Identity Management System (BPDIMS) is a conceptual design for a blockchain-based data-sharing platform aimed at being compliant with the GDPR. The blockchain stores hashes of data to verify its integrity. The system has provisions to sell data.	Dynamic	Yes	Mixed private-public	Off-chain		Proposal/Design	
Griggs et al. [57]	A system that is compliant with HIPAA and stores healthcare data off-chain, with an Ethereum blockchain recording the fact that events, like the completion of treatment, were completed. In this system, sensors communicate with smart devices, which call Ethereum smart contracts to record that the data were processed.	Not specified	Yes	Consortium	Off-chain	Ethereum	Proposal/Design	Partial - GitHub
Grishin et al. [60]	Nebula is an Exonum blockchain-based system that distributes data and computation for genomic research. Genomic data are distributed, and the blockchain serves as an index. Data owners can also control access to their information.	Grant only	Yes	Mixed private-public	Off-chain	Exonum		
Hashemi et al. [63]	A system to share health data captured by devices and sensors. It focuses extensively on giving data owners control over the data that they generate. Users receive requests and review them.	Dynamic	No		Blockchain		Proposal/Design	
Ichikawa et al. [64]	A smartphone health application that focuses on observing patterns of insomnia, using the Hyperledger Fabric blockchain to store data. The focus is on making this data tamper-proof. Users get feedback about the data that they input.	Not specified	No	Private	Blockchain	Hyperledger Fabric	Implementation	
Jiang et al. [52]	BLOCkchain-Based Platform for Healthcare Information Exchange (BlocHIE) is designed to facilitate sharing EHR and PHD. It is made up of two different blockchains - one to store Electronic Medical Records, the other for Personal Healthcare Data. Two fairness-based transaction packing algorithms are also presented.	Not specified	Yes	Public	Off-chain		Prototype	
Panetta and Cristofaro [72]	My Health My Data is an EU-funded project for dynamic consent with the aim of facilitating scientific medical research and healthcare. The blockchain is used to store consent changes.	Dynamic	No			Dynamic consent	Proposal/Design	
Rantos et al. [71]	ADVOCATE focuses on consent management of personal data collected from sensors, with a focus on being GDPR-compliant.	Dynamic	No					
Rifi et al. [55]	A system that feeds data from sensors into an off-chain database. The Ethereum blockchain stores pointers to this data. The idea is that patients can share this data with their doctors.	Not specified	Yes	Private	Off-chain	Ethereum		
Xia et al. [48, 49]	MeDShare is made up of four layers—the user layer, the data query layer, an authenticating layer, and the existing database that stores medical data. The blockchain stores a history of actions and requests. The system is aimed at removing the need for trust in medical data sharing.	Grant only	Yes		Off-chain			
Xia et al. [48, 49]	BBDS is made up of three layers—the user layer, the system management layer that contains the blockchain, and the storage layer. Medical data are stored off-chain, and the blockchain contains details about requests.	Not applicable	Yes		Off-chain			
Yue et al. [17]	Healthcare Data Gateways is a three-tiered approach for patients to share healthcare data with clinicians. First, the data are stored on the blockchain—the storage layer. Second, a data management layer restricts access to the third layer—the data usage layer.	Grant only	No	Private	Blockchain			
Zhang and Lin [62]	Each institution has its own private Juice blockchain that stores its patient data. Data to be shared among institutions is added to a consortium blockchain shared by all organizations. The system focuses on sharing data among hospitals, though tokens are still required to access a patient's data.	Not applicable	No	Mixed private-consortium	Blockchain	Juice		
Zheng et al. [70]	A conceptual design of an Ethereum-based data sharing system that is compliant with the GDPR. The system has a mobile application that collects health data from wearables and stores them in an off-chain database. The blockchain stores hashes of this data and some other, necessary metadata.	Dynamic	Yes		Off-chain	Ethereum	Proposal/Design	

Research has mainly focused on sharing Electronic Health Records (EHR) or Electronic Medical Records (EMR). The former is usually the focus in a clinical setting where medical records are used to treat patients. Commonly, these systems place data owners in control of their medical data, allowing them to share sensitive records with different institutions for improved healthcare. For example, systems like the Healthcare Data Gateway [17] and MedRec [46] let clinicians make requests for patient data. In turn, patients grant or revoke access [17, 46].

Patient control over data, combined with the blockchain’s immutable structure, makes data sharing more transparent. Al Omar et al. [47] explain that immutability makes the process more accountable. In fact, Ekblaw et al.’s MedRec goes beyond the blockchain’s structure and improves accountability and transparency by storing the access history in an always-available summary [46].

Nevertheless, the blockchain is not equipped to store copious data. As medical data grow, scalability becomes an additional consideration [48–51]. Existing blockchain solutions only store limited data in the blockchain itself, opting instead for more traditional, centralized off-chain storage approaches, with the blockchain storing only a hash of the data [46].

In this way, the blockchain’s role transforms into providing a proof-of-existence [52] or proof-of-integrity [46, 53, 54] to ensure that medical records exist and have not been tampered-with respectively. Alternatively, to preserve the utility of the blockchain, Rifi et al. [55] propose to store in it pointers to where the data actually reside.

Sharing sensitive data—or even pointers to it—in the blockchain exacerbates security and privacy concerns. To further limit access to EHR or EMR, many existing or proposed systems restrict the visibility of data. Private blockchains are the most common solution [29, 56]. Dubovitskaya et al. [56] argue that apart from restricting access to sensitive data, a permissioned blockchain is faster and does not require coming up with incentives for miners.

In some cases, private blockchains take the form of a consortium. A consortium blockchain makes data more private [57] by restricting access to it to selected organizations [58]. The Blockchain-based Personal Data and Identity Management System (BPDIMS) [59] is a proposed solution that uses multiple blockchains to share personal data—whereas the blockchain is publicly-visible, contributions to it are permissioned. Grishin et al. [60] achieve a similar model using the Exonum [61] blockchain. Conversely, Zhang and Lin’s [62] solution combines private and consortium blockchains. Each healthcare institution has its own private blockchain, but different organizations communicate data through a consortium blockchain.

Although EHR and EMR sharing has been the principal application for blockchain in healthcare, recent efforts have started shifting focus to personal health data (PHD). These systems collect and store PHD from sensors, such as wearable devices. These systems’ use extends similarly to popular healthcare solutions, enforcing access control on this data [63–65].

Genomic research too is emerging as one possible use case for the blockchain. Zhang et al.’s [66] solution stores data analyses in the blockchain. Conversely, Grishin et al.’s [60] Nebula distributes genomic data and computation, with the blockchain storing the uniform resource locator where the data are stored.

Whereas privacy concerns are on the forefront of many of the cited systems, legal issues are emerging in light of the GDPR, which came into effect in the European Union in May 2018 to protect the personal data of citizens. In biobanking, the GDPR safeguards the safety of research partners without restraining biomedical research by following two principles: transparency and accountability [67], which dynamic consent itself shares.

Nevertheless, the compliance of the blockchain with GDPR is debatable [68]. From a legal standpoint, Berberich and Steiner [69] mull the question of who the data controller is in the blockchain structure, where everyone has a copy of some or all of the data. Thus, ever since the GDPR came into effect, research has started looking at making blockchain-based systems that comply with the regulation [70, 71].

The focus of these systems is normally the use of the blockchain to store consent for how personal data should be shared, thereby enforcing individuals’ control [70, 71]. This is also the case in My Health My Data, a system that aims to make patients more aware of their data and facilitate medical data sharing for research and healthcare through dynamic consent. The system stores the dynamic consent changes in blockchain smart contracts, and as an EU-funded project, My Health My Data is especially mindful of the rights that the GDPR gives individuals [72].

Among other rights, the GDPR also allows citizens to demand the erasure of their data—the right to erasure. This possibility is seemingly incompatible with the blockchain’s immutable structure [69]. In the case of My Health My Data, which plans to store consent information in the blockchain, this characteristic clashes with the GDPR to the point that the right to erasure is not planned to be offered to patients [72]. However, the general trend of storing data off-chain helps make existing or prospective systems more compliant [59].

For example, MedRec is primarily a blockchain solution, but it stores sensitive personal information in a more traditional, centralized off-chain database from wherein data can be removed. The blockchain itself stores only hashes to this data, and a log containing patients’ permissions to clinicians to access data [46].

The USA’s Institutional Review Boards (IRB) [73] also gives many rights to human participants in research. Some of these rights are also present in the GDPR and issues like informed consent are applicable to biobanking. Choudhury et al. [29] weighed up changes to the IRB and proposed a design that would put the blockchain in agreement with it.

To the best of our knowledge, the blockchain has not been adopted yet in any dynamic consent systems in biobanking. However, just like the variety of applications in healthcare, we envisage that dynamic consent can benefit from the blockchain by not only storing the status of consent, but also by facilitating the means to audit it.

## Implementation

Dwarna is primarily a dynamic consent solution, permitting research partners to better inform and involve themselves in genomic research. However, Dwarna facilitates genomic research even in the recruitment stage. The web portal makes available to the general public governance documents and information about the Malta Biobank, as well as video interviews with research partners, relatives of research partners as well as policy advisers.

Dwarna also provides a short web form that allows members of the general public to express their interest in providing a biospecimen and becoming research partners. The form requires only the full name of the individual and contact details. An accompanying privacy policy describes how these details are used. These credentials are forwarded to the biobank manager, who uses this information to contact the individual and set up an appointment with them.

The person visits the biobank’s premises, where the biobank manager describes in detail what dynamic consent is, covering issues including procedures to be followed with incidental findings, withdrawal procedures and data sharing. At the end, if the individual agrees, they sign a consent form to bank their sample and they provide a biospecimen.

Once a biospecimen has been provided, the biobank assigns the individual a pseudonym, which is stored physically at the biobank. Subsequently, the biobank manager creates a profile for the individual in the Dwarna web portal; the login details are the pseudonym, which acts as a username, and a randomly-generated password. The profile also stores the name and contact details. The login credentials are sent to the new research partner’s email address. From that point onward, all further consenting, or withdrawal of consent, happens via the Dwarna web portal.

Once logged-in, research partners have access to another page that lists ongoing research studies. When a research partner clicks on a particular study, they are led to another page that lists information about the involved researchers, and their aims and objectives. On this same page, research partners can give consent or withdraw consent from a study. However, before giving or withdrawing consent, they need to be successful in a short test that quizzes them about their knowledge of what they are consenting to, what consenting entails and their rights as research partners.

Research partners can visit a particular research study’s page at any time to alter their consent. Within the context of Dwarna, where the initial consent to bank the biospecimen occurs during the preceding face-to-face stage at the biobank, we think of consent simply as the ‘consent change’—the research partner giving or withdrawing consent in relation to a particular study. The consent change is saved in the blockchain, which attaches a timestamp to it.

Over time, giving and withdrawing consent creates a permanent record of consent changes in the blockchain. The study page shows a timeline of the research partner’s history of consent changes to that study. In the rest of this section, we explain the technical details of how Dwarna handles this process.

### Architecture

We split the implementation of Dwarna into a frontend and a backend, with the two communicating together through a REST API, as shown in Supplementary Information (Fig. SI-1). Following open-source software best practices [74], we make Dwarna’s entire implementation available on GitHub under the GNU General Public License v3.0 [75]. Unit tests ensure the implementation’s correctness of the backend and the REST API.

The frontend is a web portal that is the main point of communication for the biobank manager, researchers, and research partners. We implement the portal using the WordPress content management system (CMS). We developed a custom WordPress plugin for the dynamic consent functionality. When users interact with the portal, Dwarna makes requests to the REST API to update information about users, studies or consent.

The backend itself is split into two components that store Dwarna’s data. First, a Hyperledger Fabric blockchain stores study identifiers and basic information about research partners, and connects them together with the research partners’ consent changes. Second, an off-chain PostgreSQL 10.10 database stores the majority of data about users and studies.

**Fig. 3:**
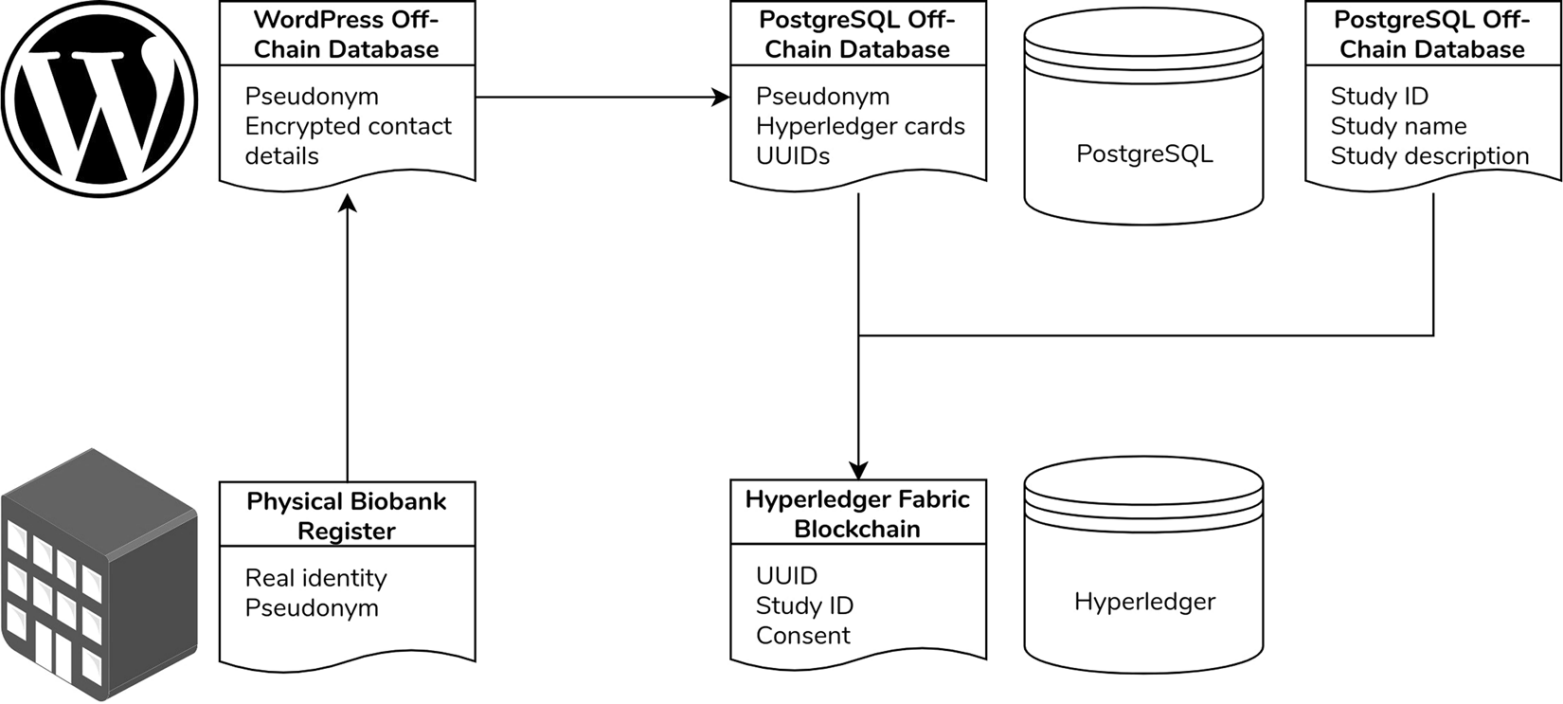
Dwarna’s data linkage. A simplified model of the research partners’ data linkage in Dwarna.

This separation of data makes it more difficult to access research partners’ data. As shown in Fig. 3, the links between the real identities of research partners and their pseudonyms are stored physically at the biobank. WordPress stores and uses these pseudonyms as login usernames. Dwarna does not use these pseudonyms to represent research partners in the blockchain, but creates instead new pseudonyms for them using universally unique identifiers (UUID) [76]. The PostgreSQL database acts as an intermediary between WordPress and the blockchain, storing the pseudonyms and related UUIDs, as well as other information about research partners and the study data.

**Fig. 4:**
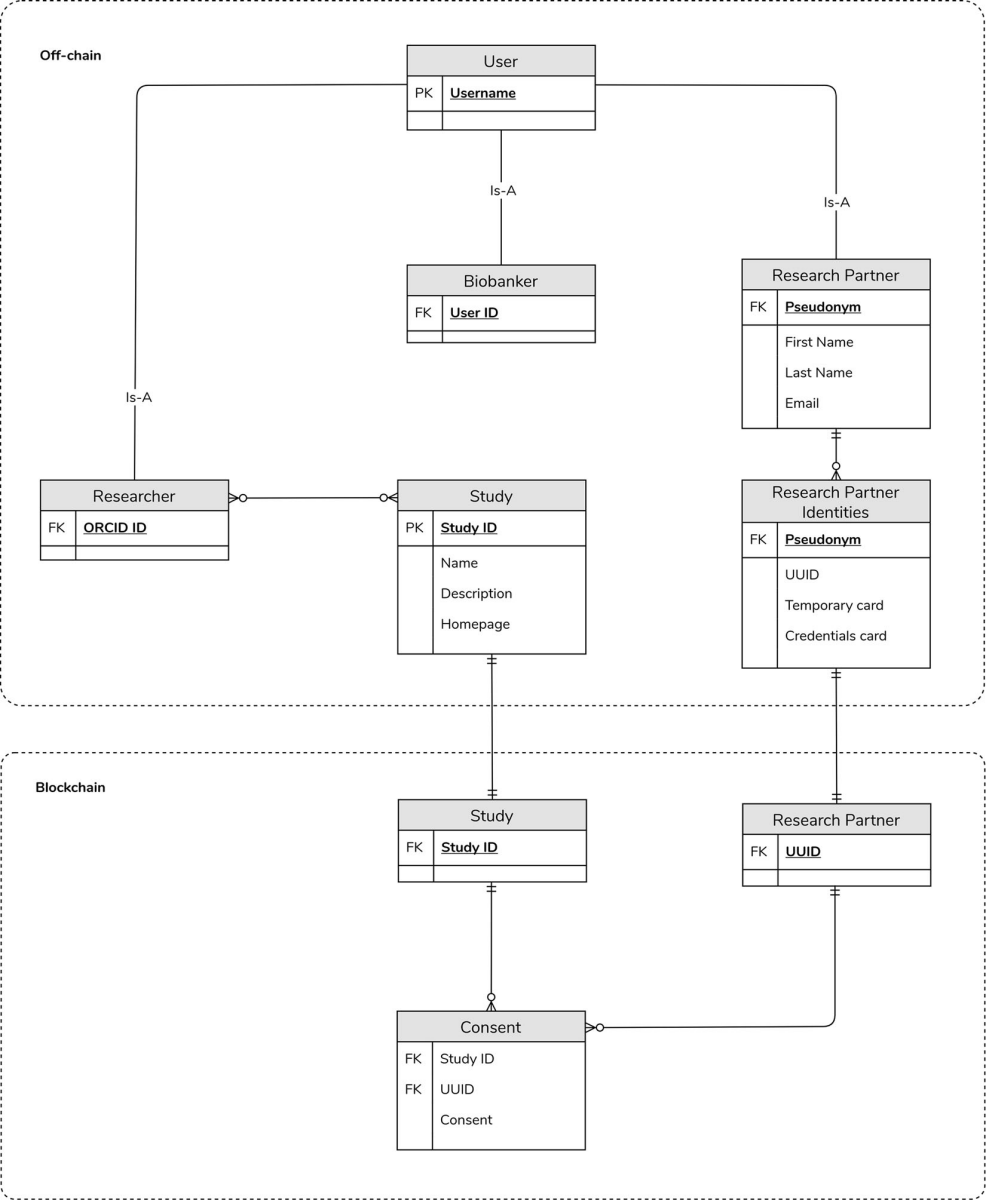
Dwarna’s ERD. An ERD that shows how Dwarna stores its data. Data about research partners, researchers, biobank managers, and studies are stored in the PostgreSQL database. Consent changes are stored in the blockchain.

The blockchain stores only consent changes as booleans that are true if the research partner gave consent, and false if they withdrew consent. The consent changes are linked with the UUID of the research partner who is altering consent and the study in question. A more detailed ERD with all of the data collected and stored by Dwarna is shown in Fig. 4.

We serve the frontend and the backend from two different servers. One server hosts the frontend and the off-chain database, whereas the other hosts the blockchain. We provide a formal analysis of this architecture as Supplementary Information. We follow the STRIDE [77] methodology to model the security threats in Dwarna, and the LINDDUN [78] methodology to model the privacy threats. These threat modeling methodologies show that Dwarna’s architecture is secure and protects research partners’ data from illicit access. We describe Dwarna’s different components in more detail in the rest of this section.

### Frontend

We serve the Dwarna portal using a standard WordPress [79] installation. The open-source CMS is among the most commonly used platforms to build websites and blogs—around a third of all websites use WordPress [80]. WordPress’ popularity makes it familiar to internet users, with a standard layout that makes it easy to use for Dwarna’s users. Furthermore, contributors have refined WordPress’ security and used its plugin architecture to create countless addons that extend the CMS’ capabilities.

Since WordPress is a CMS, the biobank may keep the general public updated about ongoing studies. Using Dwarna, users from the general public can also indicate their interest in participating in research. For this reason, the web portal also contains governance documents, videos and other information to inform prospective research partners about the biobank, ongoing scientific studies and the consenting procedures.

Users can become research partners by indicating their interest using a simple online form, which is forwarded to the biobank manager. This action opens up a direct channel of communication with the biobank staff and the recruitment process then proceeds on the biobank premises.

Once research partners give their consent to the biobank to collect their biospecimens and data, they are assigned a unique code—a pseudonym to protect their identity—and a password. Research partners can use these credentials to log in to the Dwarna web portal to access their consent trail and to modify their consent preferences—give consent to new research projects or withdraw consent from existing ones. Thus, users indicate interest in becoming research partners through the web portal, but after physically providing a biospecimen, any follow-up and re-consenting takes place on Dwarna.

The biobank-related functionality resides in a custom-built plugin written in PHP 7. In addition to members of the general public, we add three new user roles to WordPress’ user management system, each with different capabilities, as listed in Table 2.

**Table 2:** The different user roles and their capabilities in Dwarna.

Role	Capability
Biobank manager	Create, edit and remove research partners, researchers, and studies
	View a list of research partners who consented to the use of their biospecimen and data in research studies
Researcher	View aggregate data about research partners who consented to the use of their biospecimen and data in their associated studies
Research partner	Give or withdraw consent to have their data used in a research study
	View a trail of their past consent changes
	Request that the biobank destroys their biospecimen and personal data
General public	View videos, governance documents and other information about how the Malta Biobank operates
	Indicate their interest in providing a biospecimen and data to be used in research studies
	View updates about research studies on the Dwarna blog

The role of biobank managers includes creating profiles for researchers, and for research partners after they are recruited. The biobank assigns research partners a pseudonym, which Dwarna uses as a username to allow them to log in on the portal. Since the link with the real identity is stored physically at the biobank, Dwarna only ever has access to this pseudonym.

Biobank managers also create research studies, which link with the scientific investigators, or researchers. Participating researchers have visibility of aggregate data about the research partners who consented to the use of their biospecimen or data in their studies. The biobank handles the physical provision of biospecimens and data to researchers.

Research partners log in on the Dwarna portal using their pseudonym and a password to learn about ongoing research. If they are inclined to participate in any studies, they may indicate it by toggling a switch to consent. They can also withdraw that consent at any time using an identical mechanism, or demand the erasure of their data and the destruction of their biospecimen from the biobank. Research partners retain full visibility over their consent changes using their consent trail. Each study page shows a history of when and how research partners consented to participate in the associated study.

Biobank managers and researchers have access to the WordPress administration dashboard. Here too, accessibility depends on the user role; biobank managers can create researchers, research partners and studies from the plugin, whereas scientific researchers can only view aggregate data about the research partners who consented to the use of their biospecimen and data in their research studies. Our custom WordPress plugin handles all user input or requests for data, communicating it to the backend through the REST API.

### REST API

Dwarna’s REST API receives requests from the frontend, validates them, and hands them to the backend, crafting a response to the frontend. In Dwarna’s architecture, this API is written in Python 3.7, based on the python-oauth2 framework [81], and inherits the OAuth 2.0 framework’s Client Credentials grant [82]. This workflow does not require users to authenticate themselves before making requests. Instead, the frontend, which acts as Dwarna’s client in this specification, makes all requests on behalf of its users.

Following the OAuth 2.0 specification, the REST API has two roles, or servers—the authorization and resource servers. The authorization server assigns clients a unique ID and an accompanying secret, similar to the standard username-password authentication, with which to authenticate them. The resource server receives requests with access tokens and validates them. If the access token is valid and the request is well-formed, the resource server handles the request and returns a response.

The workflow to view or modify a resource starts by the frontend making requests to the authorization server for an access token. In the request, the frontend authenticates itself by providing its identifier and secret. The request also describes the access token's desired capabilities—a list of scopes—and the user on whose behalf it will be used. For example, a research partner may update their own consent, but not modify other users. If the identifier and secret match, the authorization server returns a short-lived access token that the client uses in subsequent requests to the resource server.

The resource server performs validation on requests. Most importantly, it ensures that the provided access tokens have not expired and have the necessary clearance to access a particular API endpoint. The REST API also stores the access token's owner. It uses this ownership information to restrict the scopes of requests—a research partner may only access or modify the data that belong to them. If the request satisfies all security measures, it is handed to the appropriate handler function in the backend to construct a JSON response. Otherwise, the resource server returns a failure response to the frontend.

### Backend

Dwarna’s backend incorporates two types of REST API endpoint handlers. The first set of functions operates on the blockchain solution. The second group interacts with an off-chain PostgreSQL database. The two groups remain synchronized by making calls to each other when necessary. Many changes in the frontend are reflected in the blockchain or in the off-chain database. For example, when a research partner is created in WordPress, Dwarna creates a profile in the PostgreSQL database.

As emphasized by the GDPR [83], Dwarna follows data minimization best practices to store only the necessary information about research partners. Apart from minimizing storage costs, Dwarna’s approach also limits the negative effects of data breaches or leaks. The system stores basic information about biobank managers, researchers, and research partners, as well as information about studies to which research partners may consent.

Although we follow data minimization best practices, some personal information is still necessary to be able to re-contact research partners and to allow them to reset their passwords. We store personal information in the PostgreSQL database, whereas WordPress stores the email in its database. Dwarna protects personal information and the email address using encryption. The data are decrypted by the REST API and WordPress respectively when required.

In the off-chain database, we also store additional research partner information that links them with the blockchain—credentials to update consent, described further down, and UUIDs [76] that create unique blockchain identities for research partners that are different from their biobank-assigned pseudonyms. This renders the information in the blockchain meaningless without having access to the off-chain database that links research partners’ representation in the frontend—the pseudonym—with their identities in the blockchain.

Like the PostgreSQL schema, we minimize the data stored in the blockchain. We use Hyperledger Composer [84], itself based on the Hyperledger Fabric blockchain framework [85], to host a permissioned blockchain. Apart from contributing to data security, reaching a consensus on blocks is more efficient in a permissioned Hyperledger Fabric blockchain as there is no competition for a cryptocurrency. The ERD is shown in Fig. 4.

Dwarna’s blockchain represents research partners as blockchain participants. Rather than identifying them by their biobank-assigned pseudonym, Dwarna assigns research partners UUIDs, which act as a form of new pseudonyms to represent blockchain identities. Studies are assets represented by a unique identifier assigned by the biobank manager. Dwarna also creates an asset for every single consent change, which includes not only the involved Hyperledger Fabric participant and study, but also whether consent is given or withdrawn and the timestamp when this change was effected.

In Dwarna, we deploy two Hyperledger Composer REST APIs. One is dedicated to administration tasks, such as creating research partners. The other is a multiple user API that permits research partners to make their own signed requests, including to update their consent.

To make requests to the multiple user Hyperledger Composer REST API, research partners have their own identities, which are packaged in business network cards. These cards are used by users to make their own requests, rather than have the administrator make all requests on their behalf, thereby retaining accountability.

Dwarna’s REST API takes on the role of transforming the biobank’s pseudonym into the research partner’s identities in the blockchain—the UUID. In Dwarna, the REST API creates one blockchain identity—a UUID and a business network card—for each study that a research partner participates in. When they return to withdraw consent, or to give consent to a study they previously participated in, the REST API fetches the same identity that they used in the past to consent to that study. Thus, research partners have as many identities as the number of studies that they participated in.

Before using these cards to give or withdraw consent, research partners need to authenticate with Hyperledger Composer’s multiple user REST API. Hyperledger Composer uses Passport.js to authenticate participants. We developed a custom Passport strategy that checks only whether users have logged-in on the portal and returns their username. Once they log in, Hyperledger Composer gives research partners an access token, which combines with the business network card returned by the REST API to make requests to the Hyperledger Composer multiple user REST API.

Initially, the research partners’ cards lack credentials. However, when the REST API returns the card, Dwarna automatically imports the card to their local wallet and re-authenticates to record this new card. Then, Dwarna “pings” the multiple user REST API—an action that adds credentials to the card, which is saved to the database for future reuse.

Since research partners make their own requests, Dwarna uses Hyperledger Composer’s access control language to restrict their capabilities and protect against unauthorized data access. The rules dictate that research partners can only effect consent changes on their own behalf and view their own consent changes.

With credentials added to the card, research partners can set consent by sending their access token to Dwarna’s REST API, which creates a consent asset in the blockchain. The consent asset is created asynchronously, using the provided access token so that the transaction is signed by the research partner while simultaneously allowing research partners to continue navigating Dwarna.

**Fig. 5:**
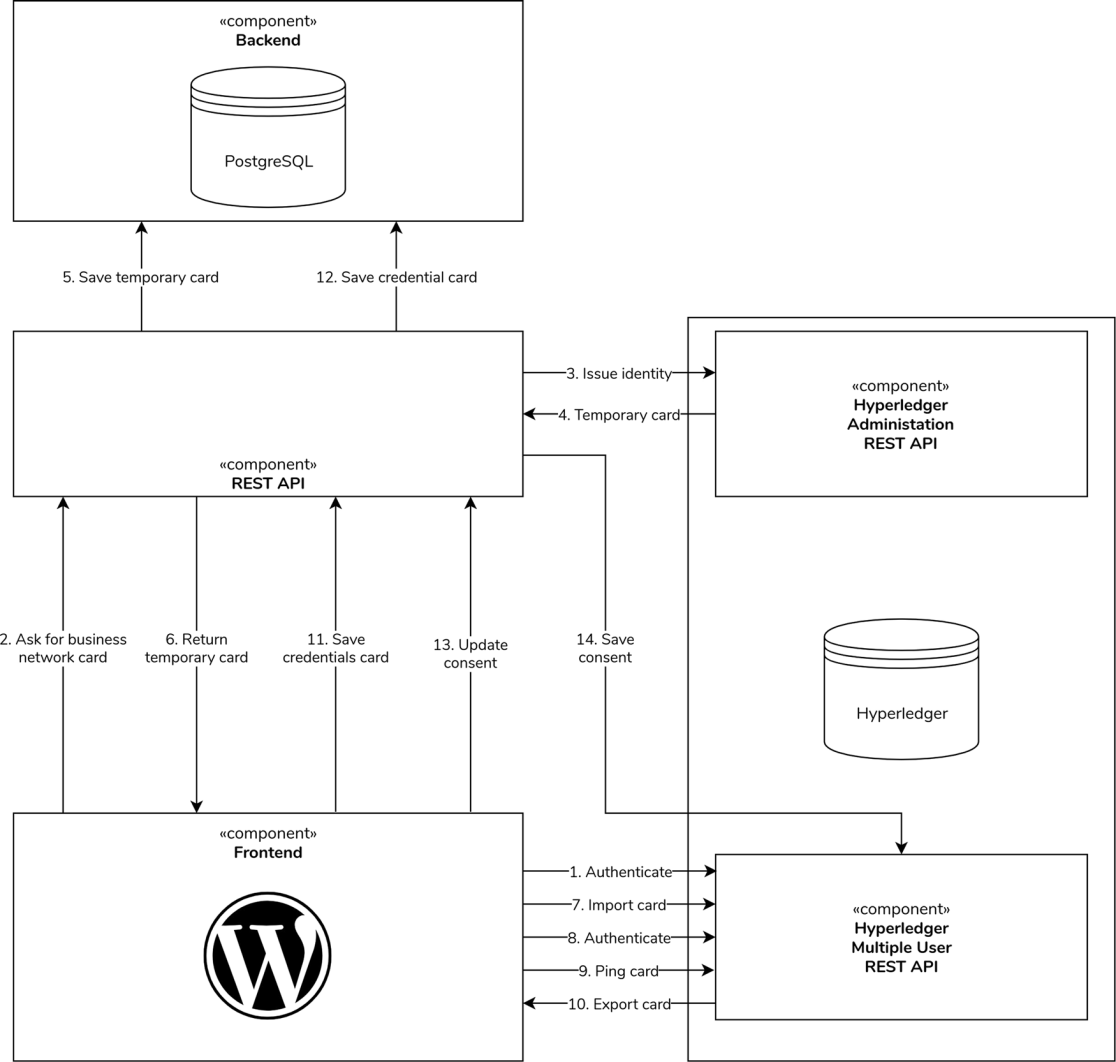
The authentication and consenting workflow. Research partners first authenticate themselves with Hyperledger Composer. Dwarna then issues identities for them if need be, saving the business network card in the off-chain database for later reuse. Research partners can then consent to research studies.

Figure 5 depicts this process, starting from a logged-in research partner authenticating with Hyperledger Composer, to getting their own identity and finally setting consent. Naturally, some steps, such as issuing new identities, are not performed when research partners have already consented to the study.

Finally, we make use of Hyperledger Composer’s query language to facilitate data requests. We use queries to fetch the consent trail of a research partner in a single study, and another that checks whether the research partner has consented to the use of their biospecimen and data in a study. The access control language propagates even to these queries—research partners may only view their own consent trails. A third query, targeted for use by administrators to fetch a list of research partners who are participating in a study, fetches all research partners’ consent changes in one study.

## Discussion

Dynamic consent aims to empower research partners to become active participants in scientific research projects. Dwarna aims to increase trust in this process by injecting transparency and accountability. Simultaneously, we keep in mind the expectations of privacy and security that surround the sharing of sensitive consent data.

Trust in the biobanking process is contingent on how the web portal stores its data. Dwarna’s data are notably separated in three different databases—consent data in the blockchain, user and study information in the off-chain PostgreSQL database, and login information in WordPress' database. Dwarna adopts data minimization best practices and stores only the necessary consent-related information.

The GDPR emphasizes the need to safeguard personal sensitive information and suggests data minimization as one way of optimizing privacy by reducing the stored data to a minimum [83]. The GDPR regulates organizations to store and process data which is considered indispensable for the research process [67]. Since the Malta Biobank itself stores and handles the biospecimens and identifiable data, Dwarna stores only the research partners' pseudonyms—the link itself is stored in a physical ledger.

Dwarna deals only with the processing of pseudonymized consent data since anonymization in biobanking is often futile; anonymization strips biospecimens and data of any identifiability with the personal identities of research partners. Thus, anonymization eliminates the possibility for research partners to request that their biospecimens and personal data are destroyed and erased respectively from the biobank.

Furthermore, biospecimens used for biomedical research are most of the time only fully effective when examined in combination with the personal medical history of the research partner. Since medical histories evolve over time and thus require updating, a link between a biospecimen stored in a biobank and the identity of the research partners is essential, as accessing medical histories would be impossible if data were to be fully anonymized.

Anonymization also eradicates the possibility of re-contacting research partners if reuse of their biospecimens and data require re-consenting. Similarly, it makes the right of research partners to withdraw consent impossible since anonymized data can never be linked back to the data subject. Consequently, full anonymity is a threat to effective genomic research and biobanking [33].

Dynamic consent does away with the need for anonymization [9] and uses pseudonymization, which is a compromise that allows for communication between stakeholders whilst protecting research partners’ privacy. The GDPR, which does not apply to anonymized data, considers pseudonymization of personal data as a safeguard in the context of genomic research as it *“can reduce the risks to the data subjects concerned and help controllers and processors to meet their data-protection obligations”* [86]. It gives research partners more control over their personal data, permits feedback and keeps researchers accountable [87].

In the Dwarna web portal pseudonymization allows us to obfuscate the link between research partners' identities and their consent. In fact, linking a research partner’s consent information with their identities requires two links. The first link is the codified connection between a research partner’s real identity and their pseudonym stored physically at the Malta Biobank. The second link is the research partners' UUID representation in the blockchain solution that is separate from the biobank's pseudonym.

We store the link between the pseudonym and the blockchain UUIDs in the off-chain PostgreSQL database, with the goal of isolating the different data of research partners. In this architecture, access to one database yields little information about individuals. Connecting consent changes to research partners’ real identities requires both links. Even if someone had to gain access to the entirety of Dwarna’s data, it would not yield the real-life identities of research partners since the link is secured physically at the biobank.

Another benefit of segregating data in the off-chain database and in the blockchain is that linkage attacks become all the more difficult. These kinds of attacks happen by combining auxiliary knowledge with the pseudonymized records to cancel out the de-identification process [88]. For example, knowing that one person in a small community has a rare disease acts as auxiliary knowledge. This information could be used to crack a person’s pseudonym if someone knows they are participating in research about this rare disease. However, our blockchain solution makes this doubly difficult.

First, the research partners’ UUIDs yield no information about their real identity; for that, access to both the off-chain and the biobank’s physical registry is necessary. Second, inferring who the research partner is through the studies that they are participating in is difficult because no study information is stored in the blockchain except a study identifier. For that too, access to the off-chain database is necessary. These two elements render linkage attacks extremely difficult without access to the off-chain database.

Having multiple UUIDs, or blockchain identities, also adds a layer of security. When research partners have multiple identities, inferring one identity does not yield information about their participation in other research studies. Given that there was sufficient external knowledge in the first place to make the first inference, then Dwarna gives away no new knowledge beyond what is already known. The only trade-off is that issuing new blockchain identities is marginally slower than re-using the same blockchain identity across all studies.

The separate storage of consent data in the blockchain also contributes to security and accountability. Our decision to host consent data on a permissioned blockchain means that it is cordoned off from the rest of the world, and through the Hyperledger Fabric blockchain, every consent transaction is permanently etched in the ledger. Moreover, as a private blockchain with no native cryptocurrency, Hyperledger Composer makes the process of committing consent changes much more efficient; unlike Ekblaw et al.’s MedRec, which uses Ethereum and its cryptocurrency [46], Dwarna does not need to come up with an incentive scheme for its miners.

Nonetheless, our decision to store data in the blockchain contrasts with the majority of existing studies that use the blockchain to handle the sharing, not storage, of EHR or EMR. However, we argue that this gives Dwarna an added layer of security. In literature, off-chain storage is often a decision driven by scalability concerns [55, 89]. Conversely, Dwarna faces few scalability issues as it stores only basic consent data that use up far less space than EHR or EMR.

By using the blockchain to store the consent changes and associate them with UUIDs, rather than the biobank’s pseudonyms, we isolate this data from the identifiable information. Since the blockchain and the off-chain database reside on different servers, using different identities for consenting renders the blockchain data almost worthless in isolation. The provision of two servers also allows for blind administrators—one for each server.

This makes all biobank operations transparent and accountable, while protecting the identities of research partners that are giving or withdrawing consent. We use this foundation to give research partners visibility of the consent process. Research partners can, at any time, audit their own consent trail from the frontend. The individual components of Dwarna protect the data of research partners by controlling access to it.

Although the blockchain’s permanence is desirable, it seems to contradict the GDPR’s right to erasure, though the regulation does acknowledge that for scientific research it may be lawful to retain the data [13]. The immutability at the core of the blockchain creates an intransigent audit trail allowing for full transparency, thus keeping data controllers accountable. In Dwarna, the difference in the representation of research partners in the off-chain and blockchain database is a compromise to bring Dwarna’s blockchain in agreement with the GDPR.

The link that connects pseudonymized research partners with their UUIDs in the blockchain resides in the off-chain database. This nonimmutable database is compliant with the GDPR and, when required, erasing the records of research partners in the off-chain database breaks the link between the biobank’s pseudonym and the research partner’s UUIDs and severs their identifiability in the blockchain. Since blockchain identities are not reused, the research partners’ consent changes become unreachable, even with linkage attacks.

A private blockchain makes linkage attacks more difficult by restricting data access. While a private blockchain contributes an elevated sense of security, it is also a compromise with transparency as it is more centralized. We rely on Hyperledger Composer’s access control and business network cards to track who made which consent changes. The web portal complements Hyperledger Composer’s protection with its own forms of security. WordPress credentials, restricted access to the OAuth 2.0-protected REST API and Hyperledger Composer’s own security combine to protect illicit access to user information. We serve these APIs over the HTTPS protocol, and lock the Hyperledger Composer administration REST API behind a firewall.

These security measures also minimize the risk of impersonation. The most obvious point of failure is gaining access to a user’s username and password. WordPress stores these passwords in hashed form using salt [90] to diminish the risk of accessing passwords through a data breach. In the future, other mechanisms, like two-factor authentication could be used to mitigate risks of accessing user credentials. The other points of failure are the database, which stores the encrypted business network cards, and the REST API. However, here too, access is governed by credentials.

Genomic research is principally about striking a balance between the risks that emerge with the provision of DNA for research and utility of the storage of such data: Oliver et al. claim that *“participants are making a deliberate privacy-utility trade-off”* [31]. The use of the blockchain within the core of Dwarna’s structure reaches the goal of injecting transparency and accountability into dynamic consent while safeguarding research partners from potentially harmful data breaches.

## Conclusion

Trust is key to the success of biobanking for genomic research and depends on robust governance procedures upholding continuous transparency and accountability. Dynamic consent empowers research partners and makes them feel valued as a part of the system that contributes actively for improved healthcare. By hosting consent changes in a Hyperledger Fabric blockchain, Dwarna makes this process transparent.

Simultaneously, Dwarna complies with the GDPR by keeping separate its data from the Malta Biobank’s own data and by following data minimization best practices. Dwarna also permits the GDPR’s right to erasure to co-exist with the blockchain while protecting research partners’ consent information.

Dwarna is open-source and can be used in scenarios where dynamic consent is required. In the future, shifting from a permissioned blockchain to a consortium blockchain could further increase transparency. In the context of biobanking, the consortium could include members of existing biobanking infrastructures, such as BBMRI-ERIC, to increase transparency and facilitate the sharing of biospecimens and data among biobanks. The implementation can also be easily adapted to perform the tasks of more traditional EHR or EMR systems; instead of associating consent with studies, the blockchain could store patient consent to share EHR or EMR data with clinicians or healthcare institutions.

Finally, a more robust model could lay the foundation for a closer collaboration between biobank managers, researchers, and research partners. A more trustworthy relationship could permit a system that uses wearable technologies to collect data from research partners on a daily basis. This data could give researchers insights about the health of research partners, facilitating biomedical research and making research partners even more actively involved.

## Supplementary information

Supplementary Information (Clean)
