# Supplementary material for: Dwarna: a blockchain solution for dynamic consent in biobanking
Source: Eur J Hum Genet. 2019 Dec 16;28(5):609–26. doi: 10.1038/s41431-019-0560-9 (PMC7170942; doi:10.1038/s41431-019-0560-9)
Supplement: Supplementary file 1 — Supplementary Information (Clean) [file 41431_2019_560_MOESM1_ESM.docx]

# Dwarna: A Blockchain Solution for Dynamic Consent in Biobanking — Supplementary Information


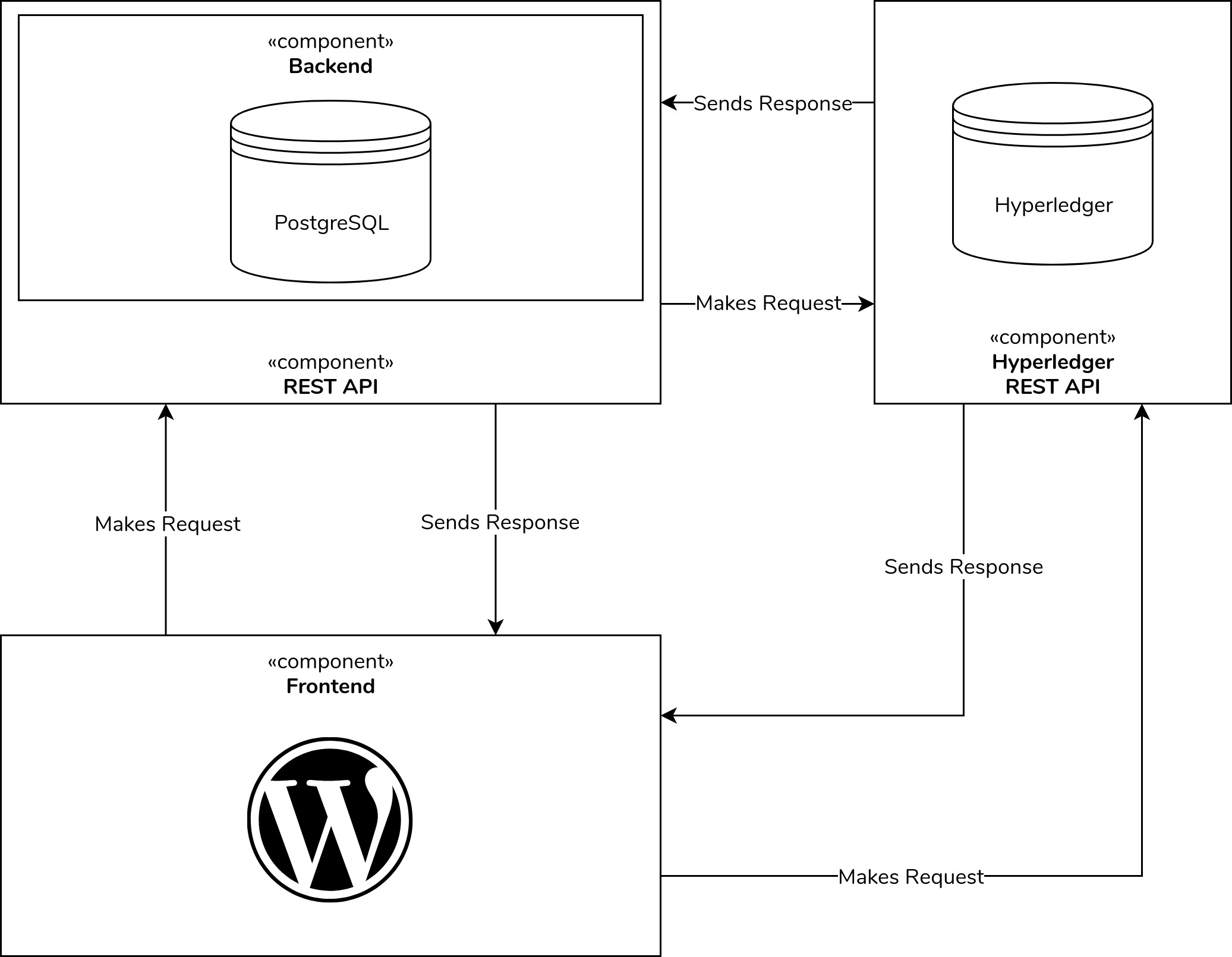

Figure SI-1: Dwarna’s architecture. The architecture is split into three main components – a WordPress-based frontend, the backend and a connecting REST API. The REST API handles requests to the backend, itself split into handlers that interface with a PostgreSQL database and a Hyperledger Fabric blockchain.


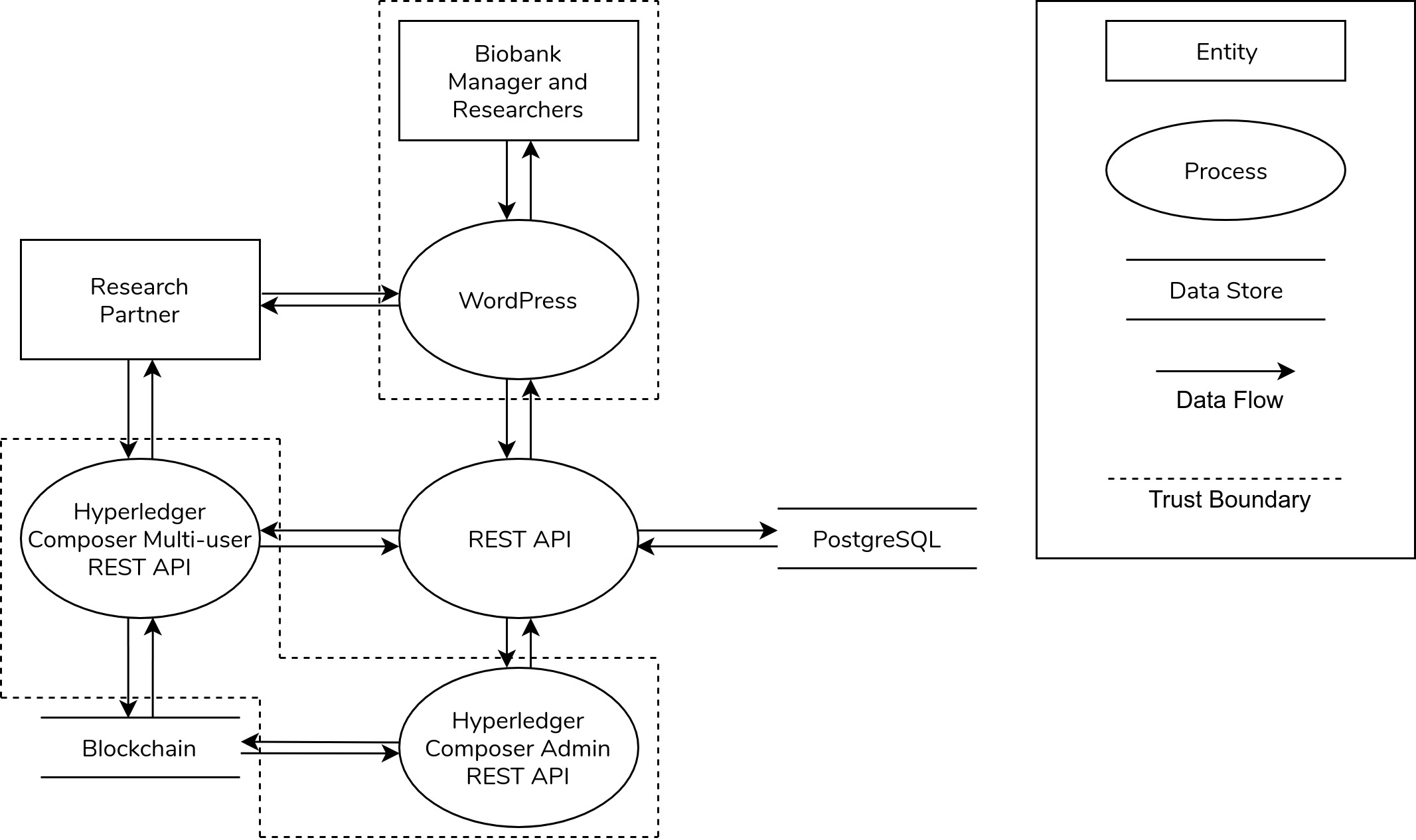


Figure SI-2: The data flow diagram of Dwarna’s architecture.

# Privacy and Security Threat Modeling (LINDDUN and STRIDE)

In the supplementary information, we describe the risks associated with the implementation of Dwarna. Firstly, we carry out a privacy analysis of Dwarna through LINDDUN. Among others, we focus extensively on how Dwarna protects the identity of research partners and how Dwarna attains accountability and transparency.

Secondly, we perform a security analysis of Dwarna through Microsoft’s STRIDE^1^. In this analysis, we delve into how Dwarna protects the research partners’ profiles and choices and explain how we ensure that the implementation is robust enough to guard against tampering and information disclosure.

## LINDDUN

In this section, we analyse Dwarna’s privacy and security through LINDDUN^2,3^. The methodology is followed retrospectively. We follow Wuyts and Joosen’s process sequentially, starting from a high-level DFD of the system. Subsequently, we model and document the threats according to the LINDDUN methodology, outlining the assumptions that we make. In the solution phase, we describe the risks of the discovered threats. Finally, we elicit the mitigation strategies to these threats and present our solutions.

### **1. DFD**

The DFD is included in Figure SI-2.

### **2. Threats**

The nodes mentioned in this subsection refer to the nodes in LINDDUN’s privacy threat trees catalog^3^.

*Assumptions*

Assumption 1: We trust many of the internal processes and their communications with the data stores. In the case of WordPress, it is an open-source Content Management System that is the fruit of the collaborative effort of a large community. Hyperledger Composer and, by implication, its REST APIs have been developed as open-source systems. However, since we developed our own REST API, we want to ensure that it is secure, alongside its data flows with the Hyperledger Composer REST APIs and the PostgreSQL database.

Assumption 2: We trust the biobank manager and the researchers, and their interactions with the portal. They are employees of the University of Malta that need access to the data and are required to abide by stringent ethical and legal regulations.

Assumption 3: We combine the linkability, identifiability and detectability of the data stores since they are tightly connected.

Assumption 4: We do not evaluate non-repudiation since Dwarna is not meant to mask actions, but its goal is to be accountable through transparency.

Assumption 5: We combine the linkability and identifiability of the data flows since they are highly similar.

Assumption 6: We ignore the detectability of data flows since we consider them to be very low threats such that they are insignificant to Dwarna.

Assumption 7: The linkability, identifiability, non-repudiation and detectability of a process are not tackled since all processes are supposed to be running and interface with the user, directly or indirectly. Their running, or knowing that they are running or have been run are not threats in and of themselves. These threats are very rare.

|  | **Threat Target** | **L** | **I** | **N** | **D** | **D** | **U** | **N** |
| --- | --- | --- | --- | --- | --- | --- | --- | --- |
| **Data Store** | PostgreSQL Database | X | X |  | X |  |  | X |
|  | Blockchain | X | X |  | X |  |  | X |
| **Data Flow** | Biobank Manager and Researchers – WordPress |  |  |  |  |  |  | X |
|  | Research Partner – Wordpress | X | X |  |  |  |  | X |
|  | Research Partner – Hyperledger Composer Multi-user REST API | X | X |  |  |  |  | X |
|  | Hyperledger Composer Multi-user REST API – Blockchain |  |  |  |  |  |  | X |
|  | Hyperledger Composer Multi-user REST API – REST API |  |  |  |  |  |  | X |
|  | WordPress – REST API | X | X |  |  |  |  | X |
|  | Hyperledger Composer Admin REST API – REST API | X | X |  |  |  |  | X |
|  | Hyperledger Composer Admin REST API – Blockchain | X | X |  |  |  |  | X |
|  | REST API – PostgreSQL Database | X | X |  |  |  |  | X |
| **Process** | WordPress |  |  |  |  |  |  | X |
|  | Hyperledger Composer Admin REST API |  |  |  |  |  |  | X |
|  | Hyperledger Composer Multi-user REST API |  |  |  |  |  |  | X |
|  | REST API |  |  |  |  |  |  | X |
| **Entity** | Research Partner | X | X |  |  |  | X |  |
|  | Biobank Manager and Researchers |  |  |  |  |  |  |  |

###

### **3. Document Threats**

*Assumptions*

Assumption 1: We ignore instances of data security that consider the *(future) receiver untrusted* (*L_df7*). The receivers in these cases are Dwarna, and eventually the biobank manager and the researchers, which are all trusted entities.

Assumption 2: We ignore contextual, but not transactional, linkability and identifiability in the data flow of the REST API. The REST API is an internal process and its related data flows are all internal – the data flows between our WordPress plugin, Hyperledger Composer’s REST APIs and our own REST API itself. Thus, contextual data is not applicable to link or identify research partners. For this same reason, the *(future) receiver untrusted (I_df5)* is also out of scope.

Assumption 3: The entity linkability cases *certificates used are too specific (L_e5)* is irrelevant to Dwarna, which uses the username-password mechanism for research partners only for logging in.

Assumption 4: *Untrustworthy receiver (L_e6)* in entity linking is irrelevant since Dwarna is the receiver.

Assumption 5: The cases of *e-id used as log-in (I_e4)* and *certificates used as login (I_e6)* are ignored as a pseudonym is used for the research partner to login. Similarly, under *pseudo-identity used as log-in (I_e5)*, the cases *token used as log-in (I_e9)* and *biometrics used as log-in (I_e10)* are irrelevant to Dwarna. In the same tree, *Untrustworthy receiver (I_e19)* is ignored as the receiver of the password is the system itself, which is trusted. We also ignore *Weak client-side storage (I_e20)* as we regard it as the research partners’ responsibility to protect their credentials.

| **Name** | **MUC 01: research partner linkability through data stores** |
| --- | --- |
| **Summary** | Research partners’ blockchain identities can be linked by gaining access to the data stores. |
| **Assets, stakeholders and threats** | Linkage attacks on research partners’ identities in the blockchain could be possible by getting access to their identities their consents. |
| **Primary misactor** | Skilled insider or outsider |
| **Basic flow** | The misactor gains illicit access to the off-chain database and collects all the blockchain identities of a research partner. This becomes more of a risk when too much data is collected, or stored for too long. |
| **Leaf Node(s)** | L_ds1, L_ds5, L_ds6 |
| **Root Node(s)** | L_ds |
| **DFD Element(s)** | Blockchain, PostgreSQL |

| **Name** | **MUC 02: research partner identification through data stores** |
| --- | --- |
| **Summary** | Research partners’ real identities could be revealed by gaining access to the off-chain database and the blockchain and performing linkage attacks. |
| **Assets, stakeholders and threats** | The research partners’ identities could be revealed by getting access to the off-chain database and the blockchain. |
| **Primary misactor** | Skilled insider or outsider |
| **Basic flow** | The misactor gains illicit access to the off-chain database and collects all the blockchain identities of a research partner, and the study information. Then, using auxiliary knowledge, such as by knowing that a person suffers from a rare disease, they perform linkage attacks to reveal the identity of a research partner’s identity through their pseudonym. |
| **Leaf Node(s)** | I_ds1, L_ds3, I_ds4, I_ds6 |
| **Root Node(s)** | I_ds |
| **DFD Element(s)** | Blockchain, PostgreSQL |

| **Name** | **MUC 03: research partner disease information detectability through data stores** |
| --- | --- |
| **Summary** | A misactor gleans that a research partner suffers from a disease by gaining access to the off-chain database and the blockchain. |
| **Assets, stakeholders and threats** | A research partners’ disease information could be detected by getting access to the data stores. |
| **Primary misactor** | Skilled insider or outsider |
| **Basic flow** | By gaining access to the blockchain and the PostgreSQL data stores, a misactor could learn the identities of the research partners in the blockchain. They would also get access to study information. Thus, the misactor could carry out a linkage attack to understand that the research partner also suffers from another disease because they consented to participate in another study. Even if the identity of a research partner is not known, knowing that they gave consent to a study in the blockchain could imply that the research partner suffers from the corresponding disease. |
| **Leaf Node(s)** | D_ds1, D_ds2 |
| **Root Node(s)** | D_ds |
| **DFD Element(s)** | Blockchain, PostgreSQL |

| **Name** | **MUC 04: research partner linkability through compromised transaction data** |
| --- | --- |
| **Summary** | The flow of transaction data is compromised by not being protected, leading to research partner linkability. |
| **Assets, stakeholders and threats** | Research partners and their actions on the portal could be linked together through weakly-protected transaction data. |
| **Primary misactor** | Outsider listening to requests. |
| **Basic flow** | The research partner makes a request and a misactor intercepts it, thereby learning information such as their pseudonym and consent to a study. Over time, they could link the pseudonym with other studies, thereby allowing them to perform linkage attacks. |
| **Leaf Node(s)** | L_df6 |
| **Root Node(s)** | L_df |
| **DFD Element(s)** | Research Partner, WordPress, Hyperledger Composer Multi-user REST API |

| **Name** | **MUC 05: research partner linkability through compromised contextual data** |
| --- | --- |
| **Summary** | The contextual data in requests could reveal some details about research partners, possibly linking them with their consent. |
| **Assets, stakeholders and threats** | Research partners and their actions on the portal could be linked together through the contextual data of the transaction. |
| **Primary misactor** | Outsider listening to requests. |
| **Basic flow** | The research partner makes a request that a misactor intercepts. In this way, over time they could analyze the requests and make deductions about the research partner. |
| **Leaf Node(s)** | L_df8, L_df9, L_df10, L_df11, L_df12, L_df13, L_df14 |
| **Root Node(s)** | L_df |
| **DFD Element(s)** | Research Partner, WordPress, Hyperledger Composer Multi-user REST API |

| **Name** | **MUC 06: research partner identification through compromised transaction data** |
| --- | --- |
| **Summary** | The flow of transaction data is compromised by not being protected, leading to research partner identification. |
| **Assets, stakeholders and threats** | The actions of research partners on the portal could lead to identification through weakly-protected transaction data. |
| **Primary misactor** | Outsider listening to requests. |
| **Basic flow** | The research partner makes a request and a misactor intercepts it, thereby learning information such as their pseudonym and consent to a study. Over time, by accruing information about pseudonyms and studies, they could perform linkage attacks to deduce the identity of the research partner. |
| **Leaf Node(s)** | I_df4 |
| **Root Node(s)** | I_df |
| **DFD Element(s)** | Research Partner, WordPress, Hyperledger Composer Multi-user REST API |

| **Name** | **MUC 07: research partner identification through compromised contextual data** |
| --- | --- |
| **Summary** | The contextual data in transactions could reveal some details about research partners, possibly linking them with their consent. |
| **Assets, stakeholders and threats** | The actions of research partners on the portal could lead to identification through the contextual data of the transaction. |
| **Primary misactor** | Outsider listening to requests. |
| **Basic flow** | The research partner makes a request that a misactor intercepts. In this way, over time they could analyze the requests and make deductions about the user, including their identity. |
| **Leaf Node(s)** | I_df8, I_df9, I_df10, I_df11, I_df12, I_df13, I_df14 |
| **Root Node(s)** | I_df |
| **DFD Element(s)** | Research Partner, WordPress, Hyperledger Composer Multi-user REST API |

| **Name** | **MUC 08: research partner linkability through compromised internal data flow** |
| --- | --- |
| **Summary** | When the data flow between the REST API and the Hyperledger Composer REST APIs or the PostgreSQL database is not fully-protected, research partner information could become linkable. |
| **Assets, stakeholders and threats** | A research partner’s data risks becoming linkable due to unsecured data flows with the REST API. |
| **Primary misactor** | Unsecured REST API |
| **Basic flow** | When the data flow between the REST API and the Hyperledger Composer REST APIs or the PostgreSQL database is not fully protected, research partner information could be used to link them with their actions. |
| **Leaf Node(s)** | L_df6 |
| **Root Node(s)** | L_df |
| **DFD Element(s)** | REST API, Hyperledger Composer Multi-user REST API, Hyperledger Composer Multi-user REST API, PostgreSQL |

| **Name** | **MUC 09: research partner identification through compromised internal data flow** |
| --- | --- |
| **Summary** | When the data flow between the REST API and the Hyperledger Composer REST APIs or the PostgreSQL database is not fully-protected, research partner information could become identifiable. |
| **Assets, stakeholders and threats** | A research partner’s data risk becoming identifiable due to unsecured data flows with the REST API. |
| **Primary misactor** | Unsecured REST API. |
| **Basic flow** | When the data flow between the REST API and the Hyperledger Composer REST APIs or the PostgreSQL database is not fully protected, research partner information could be used to identify them. |
| **Leaf Node(s)** | L_df6 |
| **Root Node(s)** | L_df |
| **DFD Element(s)** | REST API, Hyperledger Composer Multi-user REST API, Hyperledger Composer Multi-user REST API, PostgreSQL |

| **Name** | **MUC 10: research partner linkability through untrusted communication** |
| --- | --- |
| **Summary** | Untrusted communication of a research partner’s credentials could link them with their data. |
| **Assets, stakeholders and threats** | Research partners’ pseudonyms and consent changes could become linkable once a misactor obtains their credentials through untrusted communication. This happens if the misactor has access to the PostgreSQL database and to the blockchain. |
| **Primary misactor** | Skilled insider or outsider intercepts a research partner’s communication. |
| **Basic flow** | The research partner communicates with Dwarna. Unsecured communication combined with linkability in the off-chain database could link the research partners’ consent changes in the blockchain. |
| **Leaf Node(s)** | L_e3, L_e4 |
| **Root Node(s)** | L_e |
| **DFD Element(s)** | Research partner, WordPress, PostgreSQL, blockchain |

| **Name** | **MUC 11: research partner identifiability through weak credentials and untrusted communication** |
| --- | --- |
| **Summary** | The username and password of a research partner could be revealing enough such that their identity would be compromised. This, combined with untrusted communication, allows the entity to be identified. |
| **Assets, stakeholders and threats** | Research partners’ identities could be revealed in transit due to untrusted communication. |
| **Primary misactor** | Research partners could become identifiable once a misactor accrues enough information through untrusted communication or by getting access to their revealing credentials. |
| **Basic flow** | The research partner has a weak username or updates their password to something that they can easily remember, which could be identifiable. Then, when they log in, due to the communication being untrustworthy, or the storage being inadequate, the credentials, and thus the identity, leaks. |
| **Leaf Node(s)** | I_e3, I_e12, I_e17, I_e18 |
| **Root Node(s)** | I_e |
| **DFD Element(s)** | Research partner, WordPress, PostgreSQL |

| **Name** | **MUC 12: research partner unawareness** |
| --- | --- |
| **Summary** | The research partner provides too much personal data about themselves, or do not know what kind of data is stored about themselves. |
| **Assets, stakeholders and threats** | A research partner inadvertently reveals too much information about themselves, possibly including their identity. |
| **Primary misactor** | The research partner themselves. |
| **Basic flow** | The research partner is unaware of what kind of data they need to provide. Therefore they give too much information. |
| **Leaf Node(s)** | U_3, U_4, U_5 |
| **Root Node(s)** | U |
| **DFD Element(s)** | Research partner |

| **Name** | **MUC 13: tampering with consents** |
| --- | --- |
| **Summary** | A malicious actor gets access to the system and alters the consent of a research partner. |
| **Assets, stakeholders and threats** | A research partner’s consent changes are altered in a malicious act. |
| **Primary misactor** | Skilled insider or outsider. |
| **Basic flow** | A skilled insider or outsider gets access to the PostgreSQL database and to the blockchain and changes the consent of a research partner. |
| **Leaf Node(s)** | NC_1 |
| **Root Node(s)** | NC |
| **DFD Element(s)** | PostgreSQL, blockchain, research partner |

| **Name** | **MUC 14: incorrect or insufficient privacy policies** |
| --- | --- |
| **Summary** | The system does not adhere to the privacy policy. |
| **Assets, stakeholders and threats** | A research partner’s consent changes are not governed by the privacy policies. |
| **Primary misactor** | Dwarna itself. |
| **Basic flow** | Dwarna does not permit research partners from altering their consent changes, view a record of consent changes over time or request that their data be erased pursuant to the GDPR. Otherwise, Dwarna may not make it clear enough to research partners what data it collects, stores and processes. |
| **Leaf Node(s)** | NC_3, NC_4 |
| **Root Node(s)** | NC |
| **DFD Element(s)** | PostgreSQL, blockchain, research partner |

### 4. Threat Priorities

Dwarna is a dynamic, informed consent solution that aims to make the process of consenting more trustworthy. To this end, we prioritize the threats that are likely to occur and which threaten these goals, either by compromising transparency and accountability, or by not allowing research partners to be informed and involved.

We rank the threat priorities next. The likelihood is one of *highly unlikely, unlikely, possible, likely* and *highly likely*. The impact is one of *very negative, negative* and *neutral*. We regard *very negative* impact threats as those that threaten the accountability or transparency of Dwarna, or which risk identifying research partners

| **Rank** | **Name** | **Likelihood** | **Impact** |
| --- | --- | --- | --- |
| 1 | MUC 12: research partner unawareness | Possible | Negative |
| 2 | MUC 13: tampering with consents | Highly unlikely | Very negative |
| 3 | MUC 14: incorrect or insufficient privacy policies | Unlikely | Negative |
| 4 | MUC 11: research partner identifiability through weak credentials and untrusted communication | Unlikely | Very negative |
| 5 | MUC 10: research partner linkability through untrusted communication | Unlikely | Negative |
| 6 | MUC 02: research partner identification through data stores | Highly unlikely | Very negative |
| 7 | MUC 01: research partner linkability through data stores | Highly unlikely | Negative |
| 8 | MUC 03: research partner disease information detectability through data stores | Unlikely | Neutral |
| 9 | MUC 09: research partner identification through compromised internal data flow | Highly unlikely | Negative |
| 10 | MUC 08: research partner linkability through compromised internal data flow | Highly unlikely | Very negative |
| 11 | MUC 06: research partner identification through compromised transaction data | Highly unlikely | Very negative |
| 12 | MUC 04: research partner linkability through compromised transaction data | Highly unlikely | Negative |
| 13 | MUC 05: research partner linkability through compromised contextual data | Highly unlikely | Neutral |
| 14 | MUC 07: research partner identification through compromised contextual data | Highly unlikely | Neutral |

*Assumptions*

Of the above threats, we deem *MUC 05: research partner linkability through compromised contextual data* and *MUC 07: research partner identification through compromised contextual data* to be extremely unlikely with effects that are not exceedingly detrimental; the contextual data reveals little information about users. Thus, we reason that they do not warrant further examination for mitigation strategies.

### 5. Mitigation Strategies

| **MUC** | **Threat Leaf Nodes** | **Mitigation Strategy** |
| --- | --- | --- |
| MUC 12: research partner unawareness | U_3: No/insufficient feedback and awareness tools | Ensure awareness by adding feedback and awareness tools, and by making available user-friendly privacy support |
|  | U_4: No user-friendly privacy support |  |
|  | U_5: Unable to review personal information | Allow research partners to review the personal information that is stored about them |
| MUC 13: tampering with consents | NC_1: Attacker tampering with privacy policies and makes consents inconsistent | Ensure that the privacy policy is well implemented and adhered to |
| MUC 14: incorrect or insufficient privacy policies | NC_3: Inconsistent/insufficient policy management | Ensure that the privacy policy is well implemented and adhered to |
|  | NC_4: Insufficient notice |  |
| MUC 11: research partner identifiability through weak credentials and untrusted communication | I_e12: Username-password used as log-in | Apply pseudonymization so that guessing the credentials is much more difficult |
|  | I_e17: Weak password |  |
|  | I_e18: Weak username |  |
|  | I_e3: Untrusted communication | Protect the credentials of the research partner in the database |
| MUC 10: research partner linkability through untrusted communication | L_e3: Untrusted communication | Whereas the receiver – Dwarna – is trusted, the communication should happen over a secure protocol |
|  | L_e4: “fixed” login reused | Apply pseudonymization so that the login is not reused |
| MUC 02: research partner identification through data stores | I_ds1: Weak access control to database | Add access control to the database |
|  | I_ds4: Re-identification possible | Minimize the data stored in the database by removing unnecessary data |
|  | I_ds6: Linked data becomes identifiable |  |
| MUC 01: research partner linkability through data stores | L_ds1: Weak access control to database | Add access control to the database |
|  | L_ds5: Storing data too long | Minimize the data stored in the database by removing unnecessary data |
|  | L_ds6: Storing too much data |  |
| MUC 03: research partner disease information detectability through data stores | D_ds1: Weak access control to database | Add access control to the database |
|  | D_ds2: Weak information hiding | Minimize the data stored in the database by removing unnecessary data, and hide as much data as possible using encryption |
| MUC 09: research partner identification through compromised internal data flow | I_df4: Data flow not fully protected | Hide the transactional data using encryption |
| MUC 08: research partner linkability through compromised internal data flow | L_df6: Data flow not fully protected | Hide the transactional data using encryption |
| MUC 06: research partner identification through compromised transaction data | I_df4: Data flow not fully protected | Hide the transactional data using encryption |
| MUC 04: research partner linkability through compromised transaction data | L_df6: Data flow not fully protected | Hide the transactional data using encryption |

### 6. Solutions

| **MUC 12: research partner unawareness** | |
| --- | --- |
| **Mitigation Strategy** | **Solutions** |
| Ensure awareness by adding feedback and awareness tools and through user-friendly privacy support | When a user expresses interest to become a research partner, they are given ample information about what data is collected about them through a privacy policy. |
|  | When a research partner intends to give consent, show them information about what consenting means and quiz them about their knowledge. |
|  | Create an area where research partners can contact the biobank staff with queries. |
| Allow research partners to review the personal information that is stored about them | Make available the consent trail. |
|  | Store minimal data about research partners. |

###

| **MUC 13: tampering with consents** | |
| --- | --- |
| **Mitigation Strategy** | **Solutions** |
| Ensure that the privacy policy is well implemented and adhered to | The blockchain is immutable, which means that it neither permits deleting consent changes, nor creating ones without leaving a trail. |
|  | To impersonate the research partner, access to their business network cards is necessary. These cards are stored in the PostgreSQL database, which is hosted in a different server from the blockchain, thus necessitating two breaches. |

###

| **MUC 14: incorrect or insufficient privacy policies** | |
| --- | --- |
| **Mitigation Strategy** | **Solutions** |
| Ensure that the privacy policy is well implemented and adhered to | The use of the blockchain makes all consent changes transparent, with the immutable property creating a trail of consent changes. |
|  | Dwarna also offers research partners the possibility to demand that their data is erased. |
|  | Dwarna creates new pseudonyms for research partners, which are used to record consent changes in the blockchain. These pseudonyms are stored in the PostgreSQL database, which is not immutable. When research partners exercise the right to erasure, these made-up pseudonyms are deleted from the PostgreSQL database, destroying the link with the blockchain so that their consent changes in the blockchain become anonymous. |

###

| **MUC 11: research partner identifiability through weak credentials and untrusted communication** | |
| --- | --- |
| **Mitigation Strategy** | **Solutions** |
| Apply pseudonymization so that guessing the credentials is much more difficult | After providing a biospecimen, the biobank manager creates a profile for the research partner on Dwarna. This profile’s username is their biobank-assigned pseudonym, which cannot be linked to an individual’s identity without access to the biobank’s physical register. |
|  | The research partner’s first password is a strong one generated upon profile creation. Although a research partner can alter their password, this ensures that, at least initially, the credentials are secure. |
|  | All requests are made over the HTTPS protocol to encrypt all communications while logging in. |

###

| **MUC 10: research partner linkability through untrusted communication** | |
| --- | --- |
| **Mitigation Strategy** | **Solutions** |
| Whereas the receiver – Dwarna – is trusted, the communication should happen over a secure protocol | All requests are made over the HTTPS protocol to encrypt all communications. |
|  | The requests only contain the biobank-assigned pseudonym and a study identifier, which do not yield easily-understandable information about the research partner or study respectively. |
| Apply pseudonymization so that the login is not reused | The login username is the biobank-assigned pseudonym. This pseudonym is biobank-specific, and thus highly unlikely to be used elsewhere. |

###

| **MUC 02: research partner identification through data stores** | |
| --- | --- |
| **Mitigation Strategy** | **Solutions** |
| Add access control to the database | Database access is protected by a username-password combination. |
| Minimize the data stored in the database by removing unnecessary data | Pursuant to the GDPR, the database stores only the data that needs to be collected. |
|  | Personal information – the name and email addresses – is hidden through encryption. |
|  | Dwarna deals only with pseudonymization, and thus identification is only possible through linkage attacks or possibly by decrypting research partners’ information. In the blockchain, Dwarna employs its own pseudonymization, rendering linkage attacks difficult. |
|  | The PostgreSQL database and the blockchain are hosted on different servers, necessitating access to both servers for linkage attacks. |
|  | Without linkage attacks, identification relies on access to the PostgreSQL database, the blockchain and the biobank’s physical register to crack the pseudonymization. |

###

| **MUC 01: research partner linkability through data stores** | |
| --- | --- |
| **Mitigation Strategy** | **Solutions** |
| Add access control to the database | Database access is protected by a username-password combination. |
| Minimize the data stored in the database by removing unnecessary data | Pursuant to the GDPR, the database stores only the data that needs to be collected. |
|  | Where necessary, the data is hidden through encryption.  The blockchain’s pseudonymization makes linkability impossible without having access to both the PostgreSQL database and the blockchain. |
|  | The blockchain stores only the studies’ identifiers. With the study information stored in the PostgreSQL database, linkage attacks become much more difficult. |
|  | The PostgreSQL database and the blockchain are hosted on separate servers, making such linkage attacks even more difficult because they rely on two breaches. |

###

| **MUC 03: research partner disease information detectability through data stores** | |
| --- | --- |
| **Mitigation Strategy** | **Solutions** |
| Add access control to the database | Database access is protected by a username-password combination. |
| Minimize the data stored in the database by removing unnecessary data, and hide as much data as possible using encryption | Pursuant to the GDPR, the database stores only the data that needs to be collected. |
|  | Where necessary, the data is hidden through encryption. |
|  | The blockchain itself minimizes the data it stores about studies to the point that it stores only their identifiers. To link research partners with a disease, access to both the PostgreSQL database and to the blockchain is required. |
|  | To make these linkage attacks even more difficult, the PostgreSQL database and the blockchain are hosted on separate servers. |

###

| **MUC 09: research partner identification through compromised internal data flow** | |
| --- | --- |
| **Mitigation Strategy** | **Solutions** |
| Hide the transactional data using encryption | All requests are made over the HTTPS protocol to encrypt all communications. |

###

| **MUC 08: research partner linkability through compromised internal data flow** | |
| --- | --- |
| **Mitigation Strategy** | **Solutions** |
| Hide the transactional data using encryption | All requests are made over the HTTPS protocol to encrypt all communications. |

###

| **MUC 06: research partner identification through compromised transaction data** | |
| --- | --- |
| **Mitigation Strategy** | **Solutions** |
| Hide the transactional data using encryption. | All requests are made over the HTTPS protocol to encrypt all communications. |
|  | When consenting, the requests contain only the pseudonym and the study identifier. Thus, on their own, the requests do not reveal personal information. |
|  | Linkage attacks rely on persistent and continuous compromised transactions. |

###

| **MUC 04: research partner linkability through compromised transaction data** | |
| --- | --- |
| **Mitigation Strategy** | **Solutions** |
| Hide the transactional data using encryption | All requests are made over the HTTPS protocol to encrypt all communications. |
|  | When consenting, the requests contain only the pseudonym and the study identifier. Thus, on their own, the requests do not reveal personal information. |
|  | Linkability relies on persistent and continuous compromised transactions. |

## STRIDE

In the STRIDE^1^ analysis, we follow a similar workflow to LINDDUN’s. We do not present a DFD and neither a LINDDUN table. However, we document threats in the same way as LINDDUN^2^. Where available, we base this threat modeling procedure on LINDDUN’s threat catalog^3^ for STRIDE. Finally, as before, we follow LINDDUN in describing the threats’ risks and proceed directly to outlining our solutions to them.

### 1. Document Threats

The nodes mentioned in this subsection refer to the nodes in LINDDUN’s privacy threat trees catalog^3^.

*Assumptions*

Assumption 1: In spoofing, we ignore the cases of weak client-side storage (*S_13*) as we regard it as the research partners’ responsibility to protect their credentials.

Assumption 2: We combine the threats of insufficient (*S_3*) and the absence (*S_4*) of an authentication system.

Assumption 3: In the data flow tampering threat tree, we combine together all cases of violations on message integrity (*T_df1*) and channel integrity (*T_df2*).

Assumption 4: In the data store tampering threat tree, we combine the threats of bypassing the protection scheme (*T_ds1*).

Assumption 5: In the data store tampering threat tree, we ignore the sub-trees of bypassing the monitor and over-capacity failure as we deem the former irrelevant and the latter highly unlikely.

Assumption 6: We ignore the process corruption of WordPress since it is an open-source software that has been developed by a large community. Thus, corruptibility is unlikely.

Assumption 7: We combine the interactions between WordPress and the REST API, and between the Hyperledger Composer REST APIs and the REST API when evaluating the threats to process tampering. In doing so, we regard them as internal process that work towards a common goal.

Assumption 8: In *MUC 12: repudiation* we ignore the cases of spoofing as they are handled in other use cases.

Assumption 9: We ignore the information disclosure of internal data flows as they happen between trusted processes on Dwarna’s own servers. We combine data flows between the client and Dwarna’s processes as they share similar challenges. Finally, we ignore side-channel attacks (*ID_df3*) as we deem them highly unlikely.

Assumption 10: We ignore the data store’s information disclosure cases of extra-monitor access (*ID_ds3*), side-channels (*ID_ds4*) and storage management (*ID_ds5*) as they are irrelevant, highly unlikely or tangential to this threat modeling exercise.

Assumption 11: We combine the information disclosure of the WordPress and REST API processes due to the overlap between the two. We ignore *side channels (ID_p2)* as we deem them to be highly unlikely.

| **Name** | **MUC 01: spoofing by obtaining legitimate credentials from the server** |
| --- | --- |
| **Summary** | A research partner is spoofed by having their login credentials accessed from the server-side WordPress storage. |
| **Assets, stakeholders and threats** | A research partner’s credentials become known to the point that a misactor could impersonate them, possibly erasing their data or altering their consent changes. |
| **Primary misactor** | Skilled insider or outsider |
| **Basic flow** | A misactor accesses the database and retrieves the research partner’s credentials. |
| **Leaf Node(s)** | S_15 |
| **Root Node(s)** | S |
| **DFD Element(s)** | WordPress |

| **Name** | **MUC 02: spoofing through the KDC** |
| --- | --- |
| **Summary** | A research partner is spoofed by a misactor who requests an access token from the REST API on their behalf. |
| **Assets, stakeholders and threats** | The Key Distribution Center (KDC) – the REST API – is used to get access tokens on behalf of research partners, permitting the requesting user to impersonate research partners. |
| **Primary misactor** | Skilled insider or outsider |
| **Basic flow** | A misactor queries the REST API to get an access token on behalf of a research partner, thus being able to impersonate them. |
| **Leaf Node(s)** | S14 |
| **Root Node(s)** | S |
| **DFD Element(s)** | REST API |

| **Name** | **MUC 03: spoofing by obtaining the blockchain’s legitimate credentials from the server** |
| --- | --- |
| **Summary** | A research partner is spoofed in the blockchain by having their business network cards accessed from the PostgreSQL database. |
| **Assets, stakeholders and threats** | A research partner’s blockchain credentials become available to a misactor, who could impersonate them, possibly altering their consent changes. |
| **Primary misactor** | Skilled insider or outsider |
| **Basic flow** | A misactor accesses the PostgreSQL database and retrieves the research partner’s blockchain credentials. Subsequently, they can issue consent on the research partner’s behalf. |
| **Leaf Node(s)** | S_15 |
| **Root Node(s)** | S |
| **DFD Element(s)** | PostgreSQL database |

| **Name** | **MUC 04: weak transit of credentials** |
| --- | --- |
| **Summary** | A research partner is spoofed by having their credentials intercepted in transit. |
| **Assets, stakeholders and threats** | A research partners’ credentials are intercepted in transit, permitting impersonation by the misactor who gets access to them. |
| **Primary misactor** | Skilled outsider |
| **Basic flow** | A misactor intercepts communication between a client and a server while the user is logging in, thereby getting their credentials. |
| **Leaf Node(s)** | S_6 |
| **Root Node(s)** | S |
| **DFD Element(s)** | Research Partner, WordPress |

| **Name** | **MUC 05: spoofing through weak change management** |
| --- | --- |
| **Summary** | A research partner loses control over their credentials by having them changed on their behalf. |
| **Assets, stakeholders and threats** | A research partner’s credentials are overwritten by a misactor, locking the research partner out of their account, and allowing the misactor to impersonate them. |
| **Primary misactor** | Outsider |
| **Basic flow** | A misactor uses the portal to change the login credentials of another user, thereby getting access to their account and locking them out in the process. |
| **Leaf Node(s)** | S_7 |
| **Root Node(s)** | S |
| **DFD Element(s)** | Research Partner, WordPress |

| **Name** | **MUC 06: WordPress credential falsification** |
| --- | --- |
| **Summary** | The research partner’s credentials are falsified. |
| **Assets, stakeholders and threats** | A research partner’s credentials are falsified by a misactor, giving them access to the research partner’s account. |
| **Primary misactor** | Skilled outsider |
| **Basic flow** | A misactor correctly guesses the research partner’s credentials or provides equivalent credentials. |
| **Leaf Node(s)** | S_8, S_9 |
| **Root Node(s)** | S |
| **DFD Element(s)** | Research partner, WordPress |

| **Name** | **MUC 07: spoofing through insufficient or no authentication** |
| --- | --- |
| **Summary** | A research partner is spoofed through the insufficiency or outright absence of an authentication system. |
| **Assets, stakeholders and threats** | A research partner is spoofed because there is no sufficient authentication system, allowing the misactor to impersonate them. |
| **Primary misactor** | Outsider |
| **Basic flow** | A misactor exploits a weak or absent authentication system to spoof a research partner. |
| **Leaf Node(s)** | S_4, S_10, S_11, S_12 |
| **Root Node(s)** | S |
| **DFD Element(s)** | Research Partner, WordPress |

| **Name** | **MUC 08: data flow tampering** |
| --- | --- |
| **Summary** | Weak or no protection on the message or the channel is exploited to tamper with the data flow. |
| **Assets, stakeholders and threats** | A misactor exploits weak protection on a message to tamper with transactions, possibly altering consent changes or getting access to the consent trail. |
| **Primary misactor** | Skilled outsider |
| **Basic flow** | A misactor intercepts communication between the research partner and the Dwarna web portal. Exploiting either the absence of, or even weak protection of the message or channel, they tamper with the message. |
| **Leaf Node(s)** | T_df1, T_df3, T_df4, T_df5, T_df6, T_df7, T_df8, T_df9 |
| **Root Node(s)** | T_df |
| **DFD Element(s)** | Research Partner, WordPress |

| **Name** | **MUC 09: data store tampering by bypassing the protection scheme** |
| --- | --- |
| **Summary** | The data store is tampered with by bypassing the protection scheme. |
| **Assets, stakeholders and threats** | The protection scheme of the PostgreSQL database is breached by a misactor, giving them access to research partner information. |
| **Primary misactor** | Skilled outsider |
| **Basic flow** | A misactor bypasses the protection scheme, either because the protection is weak or there is none, or because of a canonalization failure. |
| **Leaf Node(s)** | T_ds4, T_ds5, T_ds6 |
| **Root Node(s)** | T_ds |
| **DFD Element(s)** | PostgreSQL |

| **Name** | **MUC 10: tampering with the REST API’s security by corrupting its state** |
| --- | --- |
| **Summary** | The REST API is corrupted by incorrect input. |
| **Assets, stakeholders and threats** | The REST API is corrupted, advertently or not, by a user who provides incorrect input. This leads to the REST API, and hence Dwarna, becoming unusable. |
| **Primary misactor** | User |
| **Basic flow** | An incorrect input by a user corrupts the REST API. |
| **Leaf Node(s)** | T_p3, T_p4 |
| **Root Node(s)** | T_p |
| **DFD Element(s)** | REST API |

| **Name** | **MUC 11: tampering with the REST API and WordPress processes through false credentials** |
| --- | --- |
| **Summary** | WordPress and the REST API are corrupted by providing false credentials. |
| **Assets, stakeholders and threats** | False credentials to WordPress or the REST API yield access to the two processes, allowing tampering with their processing. |
| **Primary misactor** | WordPress, REST API |
| **Basic flow** | The portal’s high-level processing is tampered with because of false credentials, or because the communications between WordPress and the REST API are not trusted. |
| **Leaf Node(s)** | T_p2, T_p6, T_p7 |
| **Root Node(s)** | T_p |
| **DFD Element(s)** | WordPress, REST API |

| **Name** | **MUC 12: tampering with the REST API and Hyperledger Composer REST APIs through false credentials** |
| --- | --- |
| **Summary** | The Hyperledger Composer REST APIs and the REST API are corrupted by providing false credentials. |
| **Assets, stakeholders and threats** | False credentials to the Hyperledger Composer REST APIs or the REST API yield access to the two processes, allowing tampering with their processing. |
| **Primary misactor** | Hyperledger Composer REST APIs, REST API |
| **Basic flow** | The portal’s high-level processing is tampered with because of false credentials, or because the communications between the Hyperledger Composer REST APIs and the REST API are not trusted. |
| **Leaf Node(s)** | T_p2, T_p6, T_p7 |
| **Root Node(s)** | T_p |
| **DFD Element(s)** | Hyperledger Composer REST APIs, REST API |

| **Name** | **MUC 13: repudiation in the blockchain** |
| --- | --- |
| **Summary** | Attackers’ malicious requests to the blockchain have no way of being detected and blocked. |
| **Assets, stakeholders and threats** | A misactor changes the consent changes of a research partner and covers their tracks. |
| **Primary misactor** | Skilled outsider |
| **Basic flow** | A misactor attempts to create consent changes on behalf of research partners. |
| **DFD Element(s)** | Blockchain |

| **Name** | **MUC 14: information disclosure of data flows** |
| --- | --- |
| **Summary** | The data flows between the research partner and WordPress or the Hyperledger Composer Multi-user REST API disclose information through weakly-protected messages or channels. |
| **Assets, stakeholders and threats** | By getting access to a research partner’s consent changes in transit, disease information could eventually be known, possibly gleaning the research partner’s identity using a linkage attack. |
| **Primary misactor** | Skilled outsider |
| **Basic flow** | A misactor observing the channel of communication intercepts a message that is weakly-protected. |
| **Leaf Node(s)** | ID_df4, ID_df5, ID_df6, ID_df7 |
| **Root Node(s)** | ID_df |
| **DFD Element(s)** | Research Partner, WordPress, Hyperledger Composer Multi-user REST API |

| **Name** | **MUC 15: information disclosure at PostgreSQL database** |
| --- | --- |
| **Summary** | The PostgreSQL database’s protection scheme is weak and data is stored with weak protection, leading to information disclosure. |
| **Assets, stakeholders and threats** | Research partner information is visible to a misactor who gains access to the PostgreSQL database. |
| **Primary misactor** | Skilled outsider |
| **Basic flow** | A misactor gains access to the PostgreSQL database because the protection scheme is weak. Once inside the database, weak protection of the stored research partner data leads to information disclosure. |
| **Leaf Node(s)** | ID_ds6, ID_ds7, ID_ds8, ID_ds9, ID_ds10 |
| **Root Node(s)** | ID_ds |
| **DFD Element(s)** | PostgreSQL |

| **Name** | **MUC 16: information disclosure at the Hyperledger Fabric blockchain** |
| --- | --- |
| **Summary** | The Hyperledger Fabric blockchain’s protection scheme is weak and data is stored with weak protection, leading to information disclosure. |
| **Assets, stakeholders and threats** | Research partners’ consent changes are visible to a misactor who gains access to the blockchain. |
| **Primary misactor** | Skilled outsider |
| **Basic flow** | A misactor gains access to the Hyperledger Fabric blockchain and views all of the consent changes. This could lead to linkage attacks to ultimately identify research partners. |
| **Leaf Node(s)** | ID_ds6, ID_ds7, ID_ds8, ID_ds9, ID_ds10 |
| **Root Node(s)** | ID_DS |
| **DFD Element(s)** | Blockchain |

| **Name** | **MUC 17: corruption in the REST API or WordPress** |
| --- | --- |
| **Summary** | Corruption of WordPress or the REST API leads to information disclosure. |
| **Assets, stakeholders and threats** | By corrupting WordPress the REST API, a user could receive information that is not meant for them. |
| **Primary misactor** | User |
| **Basic flow** | An incorrect call to WordPress or the REST API leads to access to memory or a failure to validate the input. In turn, the process could disclose information about research partners. |
| **Leaf Node(s)** | ID_p3, ID_p4 |
| **Root Node(s)** | ID |
| **DFD Element(s)** | WordPress, REST API, Research Partner |

| **Name** | **MUC 18: Denial of Service** |
| --- | --- |
| **Summary** | An attacker denies access to the Dwarna web portal using a denial of service attack. |
| **Assets, stakeholders and threats** | Research partners can no longer access, change or demand the erasure of their data. |
| **Primary misactor** | Malicious and skilled outsider |
| **Basic flow** | An attacker denies access to the Dwarna web portal using a denial of service attack. |
| **DFD Element(s)** | WordPress, REST API, Hyperledger Composer Multi-user REST API, Hyperledger Composer Admin REST API, Blockchain, PostgreSQL |

| **Name** | **MUC 19: elevation of privilege by corrupting the REST API** |
| --- | --- |
| **Summary** | An individual gains additional privileges through the REST API so that they can access, alter or remove research partner information. |
| **Assets, stakeholders and threats** | Research partners’ consent changes and identities could be under threat if a misactor elevates their privilege. |
| **Primary misactor** | User |
| **Basic flow** | A skilled outsider gains access to more information than they are allowed to by elevating their privileges. This could happen if the process is corrupted. |
| **Leaf Node(s)** | E_p4, E_p5 |
| **Root Node(s)** | E |
| **DFD Element(s)** | REST API |

| **Name** | **MUC 20: elevation of privilege by leveraging insufficient authorization** |
| --- | --- |
| **Summary** | A misactor leverages insufficient authorization to elevate their own privileges. |
| **Assets, stakeholders and threats** | By having elevated privileges, a misactor can gain access to research partner information, including consent changes, or alter or erase this data. |
| **Primary misactor** | Skilled and malicious outsider |
| **Basic flow** | A misactor exploits insufficient authorization in the REST API to demand elevated privileges. In doing so, they get the ability to update the consent of a research partner, erase data or view it. |
| **Leaf Node(s)** | E_p6, E_p7 |
| **Root Node(s)** | E_p |
| **DFD Element(s)** | REST API |

### 2. Threat Priorities

Similarly to the LINDDUN analysis, we rank the threat priorities. As before, the likelihood is one of *highly unlikely, unlikely, possible, likely* and *highly likely*, and the impact is one of *very negative, negative* and *neutral*. We regard *very negative* impact threats as those that threaten the accountability or transparency of Dwarna, or which risk identifying research partners.

| **Rank** | **Name** | **Likelihood** | **Impact** |
| --- | --- | --- | --- |
| 1 | MUC 13: repudiation in the blockchain | Unlikely | Very negative |
| 2 | MUC 02: spoofing through the KDC | Highly unlikely | Very negative |
| 3 | MUC 01: spoofing by obtaining legitimate credentials from the server | Highly unlikely | Very negative |
| 4 | MUC 03: spoofing by obtaining the blockchain’s legitimate credentials from the server | Highly unlikely | Very negative |
| 5 | MUC 20: elevation of privilege by leveraging insufficient authorization | Highly unlikely | Very negative |
| 6 | MUC 09: data store tampering by bypassing the protection scheme | Highly unlikely | Very negative |
| 7 | MUC 07: spoofing through insufficient or no authentication | Highly unlikely | Very negative |
| 8 | MUC 05: spoofing through weak change management | Highly unlikely | Very negative |
| 9 | MUC 11: tampering with the REST API and WordPress processes through false credentials | Highly unlikely | Very negative |
| 10 | MUC 12: tampering with the REST API and Hyperledger Composer REST APIs through false credentials | Highly unlikely | Very negative |
| 11 | MUC 06: WordPress credential falsification | Highly unlikely | Very negative |
| 12 | MUC 16: information disclosure at the Hyperledger Fabric blockchain | Highly unlikely | Negative |
| 13 | MUC 15: information disclosure at the PostgreSQL database | Highly unlikely | Negative |
| 14 | MUC 08: data flow tampering | Highly unlikely | Very negative |
| 15 | MUC 04: weak transit of credentials | Highly unlikely | Very negative |
| 16 | MUC 14: information disclosure of data flows | Highly unlikely | Negative |
| 17 | MUC 18: Denial of Service | Possible | Negative |
| 18 | MUC 10: tampering with the REST API’s security by corrupting its state | Highly unlikely | Negative |
| 19 | MUC 19: elevation of privilege by corrupting the REST API | Highly unlikely | Negative |
| 20 | MUC 17: corruption in the REST API or WordPress | Highly unlikely | Very negative |

### 3. Solutions

| **MUC 13: repudiation in the blockchain** | |
| --- | --- |
| **Solutions** | By definition, the blockchain is immutable. In this way, consent, for example, cannot be removed, but it can be updated by making a new transaction. Each such transaction is recorded in the blockchain, making it immutable. |
|  | The alternative would be to impersonate the research partner. We describe a solution in *MUC 03: spoofing by obtaining the blockchain’s legitimate credentials from the server*. |

###

| **MUC 02: spoofing through the KDC** | |
| --- | --- |
| **Solutions** | Requests to the REST API require an access token. |
|  | The REST API can be hidden behind a firewall since queries go through WordPress. |
|  | Any requests to the REST API for an access token require the client credentials, which are stored securely in the server. |

###

| **MUC 01: spoofing by obtaining legitimate credentials from the server** | |
| --- | --- |
| **Solutions** | WordPress’ MySQL database is password-protected. |
|  | WordPress does not store the passwords in plain text, but hashes them with salt. Unlike with encryption, hashes cannot be decrypted. |

###

| **MUC 03: spoofing by obtaining the blockchain’s legitimate credentials from the server** | |
| --- | --- |
| **Solutions** | To make requests to the Hyperledger Composer Multi-user REST API, it is necessary to have access to a business network card. |
|  | The business network cards are stored in the PostgreSQL database, which is hosted in a different server. |

###

| **MUC 20: elevation of privilege by leveraging insufficient authorization** | |
| --- | --- |
| **Solutions** | Once an access token is issued, the REST API does not allow it to request additional privileges. |
|  | To elevate privilege, a new access token has to be requested. |
|  | Requesting an access token is only possible through WordPress, which handles requests according to the user role, or by knowing the client credentials. These credentials are stored securely in the server. |

###

| **MUC 09: data store tampering by bypassing the protection scheme** | |
| --- | --- |
| **Solutions** | Database access is governed by a username-password protection scheme. |
|  | Access to the database can be limited behind a firewall. It is accessed only through the REST API, which resides in the same server. |

###

| **MUC 07: spoofing through insufficient or no authentication** | |
| --- | --- |
| **Solutions** | WordPress is built through the collaboration of a large open-source community, and is thus highly secure. |
|  | The usernames are the same as the biobank-assigned pseudonyms, which are not easily-guessed. |
|  | The initial passwords are randomly-generated and strong. |

###

| **MUC 05: spoofing through weak change management** | |
| --- | --- |
| **Solutions** | To change the password on behalf of the research partner, their pseudonym has to be known. This pseudonym is biobank-assigned and not easily guessed. |
|  | Changing the email address in the PostgreSQL database is not easy – the database has access control and the email addresses themselves are hidden through encryption. |
|  | WordPress’ security, which is bolstered through the collaboration of a large open-source community, is robust. The process to reset the password sends an email to the user to create a new password. |

###

| **MUC 11: tampering with the REST API and WordPress processes through false credentials** | |
| --- | --- |
| **Solutions** | The REST APIs do not permit equivalence credential falsification. |
|  | Requests to the REST API need to include an access token, which is issued by the REST API itself. |
|  | Requesting an access token is only possible through WordPress, which handles requests according to the user role, or by knowing the client credentials. These credentials are stored securely in the server. |
|  | WordPress’ security, which is bolstered through the collaboration of a large open-source community, is robust and does not permit equivalence credential falsification. |

###

| **MUC 12: tampering with the REST API and Hyperledger Composer REST APIs through false credentials** | |
| --- | --- |
| **Solutions** | The REST APIs do not permit equivalence credential falsification. |
|  | Requests to the REST API need to include an access token, which is issued by the REST API itself. |
|  | Requesting an access token is only possible through WordPress, which handles requests according to the user role, or by knowing the client credentials. These credentials are stored securely in the server. |
|  | Requests to the Hyperledger Composer REST APIs require an active business network card. These cards can only be retrieved from the PostgreSQL database. |
|  | Requests to the Hyperledger Composer REST APIs also require authentication, which is only possible through WordPress. An account is required to authenticate. |
|  | The PostgreSQL database is hosted in a different server, requiring two breaches. |

###

| **MUC 06: WordPress credential falsification** | |
| --- | --- |
| **Solutions** | Falsifying credentials is difficult since the login username is the biobank-assigned pseudonym. The username itself is not easily-guessed. |
|  | Even if the username had to be guessed, the password has to be guessed as well. In Dwarna, the initial password is a random strong one that is not tied to the research partner. |
|  | WordPress’ security, which is bolstered through the collaboration of a large open-source community, is robust and does not permit equivalence credential falsification. |

###

| **MUC 16: information disclosure at the Hyperledger Fabric blockchain** | |
| --- | --- |
| **Solutions** | Getting access to data through the Hyperledger Composer REST APIs requires access to a research partner’s business network cards – one business network card yields only information about that one individual’s participation in a single research study – or the administrator’s business network card. The former are stored in the PostgreSQL database, whereas the administrator’s business network card is stored in the server. |
|  | The PostgreSQL database is hosted on a different server than the blockchain. |
|  | The blockchain stores minimal information about both research partners and studies. This makes it difficult to understand the data without access to the PostgreSQL database. |
|  | Normal access to the Hyperledger Fabric blockchain or its REST APIs are governed by access control. The administration REST API allows more open-ended access, but it is hidden behind a firewall. |
|  | Having access to the actual blockchain data does not permit linkability as research partners’ relationships with each study are marked with a different blockchain pseudonym, or one UUID per study. |

###

| **MUC 15: information disclosure at the PostgreSQL database** | |
| --- | --- |
| **Solutions** | The PostgreSQL database has access control. |
|  | The PostgreSQL database stores minimal information about research partners. |
|  | Personal information – the names and email addresses of research partners – are hidden through encryption. |

###

| **MUC 08: data flow tampering** | |
| --- | --- |
| **Solutions** | The data flows are served over the HTTPS protocol and thus all content is encrypted. |

###

| **MUC 04: weak transit of credentials** | |
| --- | --- |
| **Solutions** | The data flows are served over the HTTPS protocol and thus all content is encrypted. |

###

| **MUC 14: information disclosure of data flows** | |
| --- | --- |
| **Solutions** | The data flows are served over the HTTPS protocol and thus all content is encrypted. |
|  | When giving consent, the data includes only the new consent state, the research partner’s pseudonym and the study identifier – data that on its own does not reveal much. |

###

| **MUC 18: Denial of Service** | |
| --- | --- |
| **Solutions** | Solutions like Cloudflare can be deployed to guard against denial of service attacks. |

###

| **MUC 10: tampering with the REST API’s security by corrupting its state** | |
| --- | --- |
| **Solutions** | The REST API performs input validation to guard against corruption. |

###

| **MUC 19: elevation of privilege by corrupting the REST API** | |
| --- | --- |
| **Solutions** | The REST API performs input validation to guard against corruption. |
|  | The REST API does not allow elevation of privilege – a new access token has to be requested with elevated privileges. |
|  | To request an access token, the client credentials need to be known. These credentials are stored on the server. |
|  | The REST API is only ever accessed from the server-side – WordPress makes the requests on behalf of front-end users – and thus can be secured behind a firewall. |

###

| **MUC 17: corruption in the REST API or WordPress** | |
| --- | --- |
| **Solutions** | Wordpress is an open-source CMS, which means that it is also robust against corruption. |
|  | Corruption in Wordpress is highly unlikely to show information beyond the logged-in user’s data. |
|  | The REST API performs input validation to guard against corruption. |

###

# References

1. Kohnfelder L, Garg P. The Threats to Our Products. Available at: https://cloudblogs.microsoft.com/microsoftsecure/2009/08/27/the-threats-to-our-products/. Accessed Sep 5, 2019.

2. Wuyts K, Joosen W. LINDDUN privacy threat modeling: a tutorial. 2015 Jul 1,.

3. Wuyts K, Scandariato R, Joosen W. LIND(D)UN privacy threat tree catalog. 2014; Available at: https://linddun.org/catalog.php.
